# Novel functional insights into ischemic stroke biology provided by the first genome-wide association study of stroke in indigenous Africans

**DOI:** 10.1186/s13073-023-01273-5

**Published:** 2024-02-05

**Authors:** Rufus O. Akinyemi, Hemant K. Tiwari, Vinodh Srinivasasainagendra, Onoja Akpa, Fred S. Sarfo, Albert Akpalu, Kolawole Wahab, Reginald Obiako, Morenikeji Komolafe, Lukman Owolabi, Godwin O. Osaigbovo, Olga A. Mamaeva, Brian A. Halloran, Joshua Akinyemi, Daniel Lackland, Olugbo Y. Obiabo, Taofik Sunmonu, Innocent I. Chukwuonye, Oyedunni Arulogun, Carolyn Jenkins, Abiodun Adeoye, Atinuke Agunloye, Okechukwu S. Ogah, Godwin Ogbole, Adekunle Fakunle, Ezinne Uvere, Motunrayo M. Coker, Akinkunmi Okekunle, Osahon Asowata, Samuel Diala, Mayowa Ogunronbi, Osi Adeleye, Ruth Laryea, Raelle Tagge, Sunday Adeniyi, Nathaniel Adusei, Wisdom Oguike, Paul Olowoyo, Olayinka Adebajo, Abimbola Olalere, Olayinka Oladele, Joseph Yaria, Bimbo Fawale, Philip Ibinaye, Olalekan Oyinloye, Yaw Mensah, Omotola Oladimeji, Josephine Akpalu, Benedict Calys-Tagoe, Hamisu A. Dambatta, Adesola Ogunniyi, Rajesh Kalaria, Donna Arnett, Charles Rotimi, Bruce Ovbiagele, Mayowa O. Owolabi, for the SIREN Team

**Affiliations:** 1 Institute for Advanced Medical Research and Training, College of Medicine, University of Ibadan, Ibadan, Nigeria; 2 Center for Genomic and Precision Medicine, College of Medicine, University of Ibadan, Ibadan, Nigeria; 3 Department of Medicine, College of Medicine, University of Ibadan, Ibadan, Nigeria; 4 Department of Biostatistics, University of Alabama at Birmingham, Birmingham, AL USA; 5 Department of Medicine, Kwame Nkrumah University of Science and Technology, Kumasi, Ghana; 6 Department of Medicine, University of Ghana Medical School, Accra, Ghana; 7 Department of Medicine, University of Ilorin Teaching Hospital, Ilorin, Nigeria; 8 Department of Medicine, Ahmadu Bello University, Zaria, Nigeria; 9 Department of Medicine, Obafemi Awolowo University Teaching Hospital, Ile-Ife, Nigeria; 10 Department of Medicine, Aminu Kano Teaching Hospital, Kano, Nigeria; 11 Jos University Teaching Hospital, Jos, Nigeria; 12 Department of Epidemiology, School of Public Health University of Alabama at Birmingham, Birmingham, USA; 13 Department of Pediatrics, Volker Hall University of Alabama at Birmingham, Birmingham, USA; 14 Department of Epidemiology and Medical Statistics, College of Medicine, University of Ibadan, Ibadan, Nigeria; 15 Medical University of South Carolina, Charleston, SC USA; 16 Delta State University/Delta State University Teaching Hospital, Oghara, Nigeria; 17 Department of Medicine, Federal Medical Centre, Ondo State, Owo, Nigeria; 18 Department of Medicine, Federal Medical Centre Umuahia, Abia State, Umuahia, Nigeria; 19 Department of Health Education, Faculty of Public Health, University of Ibadan, Ibadan, Nigeria; 20 Department of Public Health, College of Health Sciences, Osun State University, Osogbo, Nigeria; 21 Genetics and Cell Biology Unit, Department of Zoology, Faculty of Science, University of Ibadan, Ibadan, Nigeria; 22 Department of Food and Nutrition, Seoul National University, Seoul, South Korea; 23 Department of Medicine, Federal Medical Centre, Abeokuta, Nigeria; 24 Weill Institute for Neurosciences, School of Medicine, University of California San-Francisco, San Francisco, USA; 25 Federal Teaching Hospital, Ido-Ekiti, Ekiti State Nigeria; 26 Translational and Clinical Research Institute, Newcastle University, Newcastle upon Tyne, United Kingdom; 27 Department of Epidemiology and Biostatistics, Arnold School of Public Health, University of South Carolina, Columbia, USA; 28 Center for Genomics and Global Health, National Human Genome Research Institute, NIH, Bethesda, USA; 29 University College Hospital, Ibadan, Nigeria; 30 Lebanese American University of Beirut, Beirut, Lebanon; 31 Blossom Specialist Medical Center, Ibadan, Nigeria

**Keywords:** Stroke, Genomics, GWAS, African ancestry, Ischemic stroke, SNP, miRNA

## Abstract

**Background:**

African ancestry populations have the highest burden of stroke worldwide, yet the genetic basis of stroke in these populations is obscure. The Stroke Investigative Research and Educational Network (SIREN) is a multicenter study involving 16 sites in West Africa. We conducted the first-ever genome-wide association study (GWAS) of stroke in indigenous Africans.

**Methods:**

Cases were consecutively recruited consenting adults (aged > 18 years) with neuroimaging-confirmed ischemic stroke. Stroke-free controls were ascertained using a locally validated Questionnaire for Verifying Stroke-Free Status. DNA genotyping with the H3Africa array was performed, and following initial quality control, GWAS datasets were imputed into the NIH Trans-Omics for Precision Medicine (TOPMed) release2 from BioData Catalyst. Furthermore, we performed fine-mapping, trans-ethnic meta-analysis, and in silico functional characterization to identify likely causal variants with a functional interpretation.

**Results:**

We observed genome-wide significant (*P*-value < 5.0E−8) SNPs associations near *AADACL2* and miRNA (*MIR5186*) genes in chromosome 3 after adjusting for hypertension, diabetes, dyslipidemia, and cardiac status in the base model as covariates. SNPs near the miRNA (*MIR4458*) gene in chromosome 5 were also associated with stroke (*P*-value < 1.0E−6). The putative genes near *AADACL2*, *MIR5186*, and *MIR4458* genes were protective and novel. SNPs associations with stroke in chromosome 2 were more than 77 kb from the closest gene *LINC01854* and SNPs in chromosome 7 were more than 116 kb to the closest gene *LINC01446* (*P*-value < 1.0E−6). In addition, we observed SNPs in genes *STXBP5-AS1* (chromosome 6), *GALTN9* (chromosome 12), *FANCA* (chromosome 16), and *DLGAP1* (chromosome 18) (*P*-value < 1.0E−6). Both genomic regions near genes *AADACL2* and *MIR4458* remained significant following fine mapping.

**Conclusions:**

Our findings identify potential roles of regulatory miRNA, intergenic non-coding DNA, and intronic non-coding RNA in the biology of ischemic stroke. These findings reveal new molecular targets that promise to help close the current gaps in accurate African ancestry-based genetic stroke’s risk prediction and development of new targeted interventions to prevent or treat stroke.

**Supplementary Information:**

The online version contains supplementary material available at 10.1186/s13073-023-01273-5.

## Background

Stroke has the largest racial disparity of any chronic disease with a striking disparity in the burden of stroke among individuals of African ancestry compared to other populations [1–5]. However, the genetic architecture of stroke in indigenous African populations is largely unknown [1, 6, 7]. Previous genome-wide association studies (GWAS) have identified important genetic variants associated with stroke risk in European and Asian ancestry populations, with sparse inclusion of African-American populations [8–11] (who have up to 80% African genetic admixture) [12, 13]. Despite this progress, the stroke genetic landscape remains incomplete. It is imperative to explore indigenous African populations because of the higher stroke heritability in African ancestry populations [14, 15]. The increased diversity of the African genome [16, 17] also improves the potential for making novel discoveries [18]. Moreover, the inclusion of African ancestry populations is vital to trans-ancestry meta-analysis with implications for fine-mapping of known stroke-associated loci, uncovering of novel loci, characterization of causal variants, design of polygenic risk scores, development of new targeted therapies, and personalized interventions for stroke in Africans and other global populations.

In a GWAS meta-analysis of stroke in > 22,000 individuals of African ancestry undertaken by the Consortium of Minority Population GWAS of Stroke (COMPASS)) (physician-adjudicated stroke patients = 3734 and no history of stroke = 18317), one single-nucleotide polymorphism (SNP rs55931441) near the *HNF1A* gene attained genomic significance, while variants in 24 additional unique loci including the *SFXN4* and *TMEM108* genes demonstrated suggestive associations [8]. In the most recent GIGASTROKE project which involved cross-ancestry GWAS meta-analyses of stroke and its subtypes in 110,182 stroke patients (33% non-European) and 1,503,898 control individuals from five ancestries, association signals were detected at 89 independent loci, and effect sizes were correlated across ancestries demonstrating consistent directionality even when significance was not attained. New drug targets were discovered [19]. However, no variants were described for indigenous African populations.

The Stroke Investigative Research and Education Network (SIREN) is the largest epidemiological study on stroke among indigenous Africans with dual goals of characterizing the dominant modifiable vascular risk factors [20] and unraveling potential unique genetic variants associated with stroke occurrence among West Africans. Herein, we report the findings of the first stroke GWAS performed in an indigenous African population of 3434 subjects (1691 ischemic stroke cases and 1743 stroke-free controls) from the SIREN Study. The report also includes an African ancestry meta-analysis combining summary statistics from the COMPASS Consortium (*n* > 22,000; 3734 cases, 18,317 controls) [8, 9] and a trans-ancestry meta-analysis with summary datasets from the MEGASTROKE [10] (521,612 individuals: 67,162 cases and 454,450 controls). We fine-mapped identified GWAS loci using PAINTOR. To understand the functional relevance of putative genes, we functionally annotated potential causal variants through the Cerebrovascular Disease Knowledge Portal [21] (https://cd.hugeamp.org/), the GTEx Portal (https://www.gtexportal.org), and chromatin interaction and eQTL analysis using Functional Mapping and Annotation of Genome-Wide Association Studies (FUMA) [22, 23]. Additionally, we used the University of California, Santa Cruz (UCSC) browser to confirm the potential chromatin interactions in putative genes.

## Methods

### Patient enrollment and data acquisition

The rationale and design of the SIREN study have been described elsewhere [24]. In brief, the SIREN study was initiated in August 2014 as a multi-center case-control study with 16 sites in Nigeria and Ghana. The ethnographic characteristics of the study population are as previously described [25]. Ethical approval was obtained for all study sites, and informed consent was obtained from all subjects. Cases were consecutively recruited consenting adults (aged 18 years or older) with first clinical stroke within 8 days of current symptom onset or “last seen without a deficit” with confirmatory cranial CT or MRI scan performed within 10 days of symptom onset. Stroke-free controls were also recruited, and their status ascertained with a locally validated version of the Questionnaire for Verifying Stroke-Free Status (QVSFS) [26].

Relevant data were collected, including basic demographic and lifestyle data (ethnicity, native language of the subjects and their parents, socioeconomic status, dietary patterns, routine physical activity, stress, depression, cigarette smoking, and alcohol use). Cardiovascular and anthropometric measurements were obtained using standard techniques, and neurologic assessment was carried out to assess neurologic deficits and ascertain stroke severity using the National Institute of Health Stroke Severity Score. Blood samples were collected from all subjects at baseline for determination of parameters including fasting lipid profile, blood glucose, and HbA1c. Stroke diagnosis and phenotyping were undertaken as previously described [20]. Determination of stroke etiology (large vessel, small vessel, cardioembolic and undetermined) using the Trial of Org 10172 in Acute Stroke Treatment (TOAST) criteria (single dominant causative classification) was via a rigorous process of investigative evaluation including neuroimaging (CT/MRI), 12 – lead electrocardiography, echocardiography, and carotid doppler ultrasonography as previously described [20, 24].

### Description of risk factors

Hypertension was defined as sustained systolic BP > 140 mmHg or diastolic BP > 90 mmHg after the onset of stroke, a history of hypertension, or taking antihypertensive medications before the stroke [20]. Diabetes mellitus was defined based on the previous history of diabetes mellitus, use of medications for diabetes mellitus, fasting glucose levels > 126 mg/dl, and/or HBA1c > 6.5% [20]. Dyslipidemia was defined following the recommendations of the US National Cholesterol Education Program as a high fasting serum total cholesterol > 200 mg/dl or high-density lipoprotein (HDL) < 40 mg/dl [6] or low-density lipoprotein (LDL) > 130 or triglyceride (Trig) ≥ 150 mg/dl or history of use of statins before the stroke. Cardiac disease was defined as a history or current diagnosis of atrial fibrillation, cardiomyopathy, heart failure, ischemic heart disease, and rheumatic heart disease. Obesity was assessed by defining central adiposity using waist-hip ratio. A waist-to-hip ratio of ≥ 0.90 (men) and ≥ 0.85 (women) was reported as Yes, while values below this were reported as No [6, 20, 24].

### Genotyping and imputation

The samples included in this study were genotyped using Illumina’s H3Africa microarray chip. Using Illumina’s GenomeStudio software and its data management plugins, the raw genotypes data was converted into PLINK formatted datasets to interoperate with the downstream quality control (QC) and statistical analysis. Sample QC excluded (a) individuals with sex discordance between reported and observed from genetic data, (b) cases with hemorrhagic stroke, (c) duplicate sample pairs after validating similarity in genetic data based on > 90% concordance in genotype data, (d) mixed-up samples based on genotypic concordance between samples, and (e) outlier samples through estimation of genetic principal components. To address potential population stratification, we performed principal component (PC) analysis using EIGENSTRAT’s Smartpca module [27, 28]. We also excluded participants whose phenotypic and genetic data did not pass quality control and had missing variables in any covariates.

There were 2,221,421 raw variants processed through a series of in-house QC steps, including (a) retention of autosomal SNPs only, (b) removal of ambiguous SNPs (A/T and C/G), (c) removal of non-biallelic variants (e.g., indels, SNPs without a valid alternative allele in the bim file for example “0/T”), and (d) handling strand inconsistencies. Furthermore, SNPs were removed for violation of Hardy-Weinberg equilibrium *P* < 1.0E−05, minor allele frequency (MAF) < 1%, and/or a missing rate > 10%. After implementing these steps, 1,815,856 genotyped variants were included for imputation. In addition to the above-mentioned QC metrics, McCarthy Group Tools (https://www.well.ox.ac.uk/~wrayner/tools/) was employed to handle strand inconsistencies, ref/alt allele assignment, removal of SNPs not in reference panel, and filtering out SNPs with out-of-bound differences in the minor allele frequency (MAF) when compared with 1000Genomes African-Americans (i.e., SNPs with > 0.2 allele frequency difference between the SIREN cohort and 1000 genomes). Allele frequency and allele assignment fixes in McCarthy tools were performed based on the population-specific reference panels to ensure the African cohort of the SIREN study was compared with its corresponding sub-population cohort of the 1000 genomes.

Having a well-curated quality reference panel is key to discovering true biological signals and minimizing false positives or negatives in our genome-wide association studies. We used the TOPMed release2 reference panel from the BioData Catalyst (https://imputation.biodatacatalyst.nhlbi.nih.gov/#!) for imputing the genotypes. The TOPMed Version release2 panel comprised 97,256 samples and 308,107,085 genetic variants distributed across the 22 autosomes and the X chromosome inferred from jointly called variant set derived from whole-genome sequencing of TOPMed samples. TOPMed Imputation server was configured to (a) use TOPMed as the reference panel, (b) retain variants with an imputation quality filter (*R*^2^) > 0.3, (c) employed Eagle v2.4 [29](Ref) for phasing, (d) QC frequency check was conducted before imputation, and (e) Quality Control and Imputation mode was enabled for output QC stats along with imputed dosage and info datasets. Upon completion of imputation to the TOPMed *R*^2^ (Freeze8) panel, variants were retained if (a) the imputation quality (*R*^2^) > 0.3 and (b) the minor allele count (MAC) > 20. Variants with imputed genotype probabilities < 0.9 were masked as missing to ensure high-quality calls.

Before exploring the association between imputed SNPs and predictors of interest, imputed variants were further quality controlled for genotypic characteristics. SNPs were retained for association analysis only when they met the criteria of (a) attaining a Minor-Allele Frequency > 1%; (b) being SNPs only, not indels (which were removed); and (c) having an Imputation quality, *R*^2^ > 0.3. Although imputation quality is a composite score that would aggregate individual genotype quality across all samples and issue a variant level metric, to foster high-quality genotype calls, we examined the genotype probabilities (GP) associated with each genotype call and masked the genotype calls to missing if the probability of the inferred call was < 90%. All post-imputation quality control steps were conducted using PLINK 1.9 [30] and VCFTOOLS 0.19 (https://vcftools.sourceforge.net/man_latest.html). After imputation, a total of 50,877,079 variants were processed through a quality-control pipeline to yield a final count of 44,159,966 variants (*R*^2^≥0.3) for statistical association tests in PLINK1.9. Of the 44,159,966 SNPs used for downstream association analysis, 77% of imputed variants had *R*^2^≥0.8, and 91% had *R*^2^≥0.5.

### Association methods and analyzed models

Statistical association analysis was conducted using PLINK 1.9. To test for associations between ischemic stroke status and variant SNPs, we fitted a logistic regression model where SNP was modeled as a predictor variable whose values were equal to the number of copies of the minor allele (0, 1, 2) (i.e., additive mode of inheritance). In all association analyses, we used the first 10 principal components (PCs) as covariates to control for ancestry. Our primary model (model 0) for association included sex, age, 10 PCs, and SNP as a covariate in logistic regression. For sensitivity analyses, the stroke risk factors were added to the base model in nested regression models hierarchically to ensure the significant SNPs found in the base model are associated with stroke and are not mediated by risk factors. The sensitivity analysis models are given below:

- Model 1: stroke status ~ sex + age + PCs1 … 10 + SNP + hypertension
- Model 2: stroke status ~ sex + age + PCs1 … 10 + SNP + hypertension + diabetes
- Model 3: stroke status ~ sex + age + PCs1 … 10 + SNP + hypertension + diabetes + dyslipidemia
- Model 4: stroke status ~ sex + age + PCs1 … 10 + SNP + hypertension + diabetes + dyslipidemia + cardiac disease status
- Model 5: stroke status ~ sex + age + PCs1 … 10 + SNP + hypertension + diabetes + dyslipidemia + cardiac disease status + waist-hip ratio

### Other cohorts

The COMPASS and MEGASTROKE were also involved in the analysis. The constituent studies of both COMPASS and MEGASTROKE are described in Additional file 2: Other Study Cohorts.

### Meta-analysis

We meta-analyzed association test results using the random-effects model of Han and Eskin implemented in METASOFT [31] with SIREN and COMPASS data sets. Lastly, we used Meta-Analysis of TRansethnic Association studies (MANTRA) [32] software to perform meta-analysis using SIREN (a West-African study), COMPASS (an African-American study), and MEGASTROKE (a European study). There are several advantages of using METASOFT, namely, (1) it provides fixed effects model (FE) based on inverse-variance-weighted effect size similar to METAL [33], (2) conventional random effects model (RE) based on inverse-variance-weighted effect size, (3) Han and Eskin’s random effects model (RE2) optimized to detect associations under heterogeneity, and (4) binary effects model (BE) optimized to detect associations when some studies have an effect and some do not have any effect.

### Fine-mapping

In our fine-mapping analysis, we used the PAINTOR [34] software package to discover potential causal variants. Although fine-mapping regions are defined as regions identified using a window (~50 kb) around the most significant variant; given the distribution of intergenic variants with genome-wide association significance of *P*-value < 1.0E−4, we expanded to a wider window where variants’ linkage disequilibrium with the lead variant extended outside the window. This was achieved by manual inspection of regional association plots to ensure the most relevant region was adequately captured.

To determine top tissue-based annotation sets for each region, we used the approach showcased in the PAINTORv3 fine-mapping software distributed through the GitHub repository. To determine the annotation relevant to stroke, we ran PAINTOR on each annotation independently. The sum of the log-Bayes factors (BFs) and effect size estimates for each annotation is further converted to relative probability for an SNP to be causal in a certain annotation track. To test the significance of annotation, the sum of the log-Bayes factors with only baseline annotation was compared with both baseline and the annotation of interest. The significance of the enrichment was further calculated from a standard ratio test comparing null (baseline annotation) and alternate (both baseline and annotation of interest) modes. By the likelihood ratio test (LRT) approach of testing each annotation, we selected the top 10 annotations to calculate the posterior probability of each SNP within our sliding window containing top GWAS SNPs.

### Functional stratum of significant hits

The working set of top SNPs from our association analysis was further annotated using ANNOVAR to determine both gene and SNP level function. dbSNP151 data release from UCSC was employed to assign rs# naming conventions to our variants reported in the additional file results dataset. To address discrepancies in the genome geography between human genome builds hg19 and hg38, functional annotations for both hg19 and hg38 are catalogued in all additional file tables. Since the traditional annotation assignment is based on just the genomic transcription coordinates of a gene, an additional 50 kb flanking distance was allowed for top SNPs to finalize the gene assignment to association analysis top SNPs. An arbitrary flanking distance of 50 kb around the transcription start and end positions allows reporting SNPs with significant association with ischemic stroke that could circumscribe broader biochemical signatures typically associated with non-coding functional elements like gene promoters, upstream enhancers, regulators, insulators, and TFBS (transcription factor binding sites).

### Functional mapping and annotation (FUMA)

FUMA [22] is an online platform for the functional mapping of genetic variants. FUMA performs functional annotation of GWAS results, prioritization of potential causal genetic variants and genes, and interactive visualization by biological data repositories and tools. FUMA contains two core functions to annotate input summary statistics (both SNPs and genes) to prioritize potential causal genetic variants and genes: SNP2GENE and GENE2FUNC modules. In the SNP2GENE module, SNPs are annotated with their biological function and mapped to genes based on positional and functional information of SNPs. Functionally annotated SNPs are mapped to genes based on functional consequences on genes (positional mapping), expression quantitative trait loci (eQTLs), and chromatin interactions of phenotype relevant tissue types. FUMA utilizes three strategies. First is positional mapping based on the physical distances (within a 10-kb window) from known protein coding genes in the human reference assembly (GRCh37 or hg19). Second is eQTL mapping with capturing information from three data repositories (GTEx, Blood eQTL browser, and BIOS QTL browser) and mapping SNPs to genes based on a significant eQTL association. It should be noted that eQTL mapping is based on cis-eQTLs (local regulatory effect within 1 Mb). A false discovery rate (FDR) of 0.05 is used to define significant eQTL association. Third is chromatin interaction mapping, involving mapping of SNPs to the promoter regions of genes based on significant chromatin interactions. FUMA selects chromatin interactions for which one region involved in the interaction overlapped with predicted enhancers and the other overlapped with predicted promoters 250 bp upstream and 500 bp downstream of the transcription start site (TSS) of a gene. By combining these three mapping strategies, FUMA prioritizes genes that are most likely to be involved in the trait of interest such as ischemic stroke. To obtain insight into putative causal mechanisms, the GENE2FUNC process annotates the prioritized genes in biological context, such as tissue specific gene expression pattern, and enrichment of gene sets.

### Gene set analysis

Genes implicated by mapping of GWAS SNPs were further investigated using the GENE2FUNC procedure in FUMA, which provides hypergeometric tests of enrichment of the list of mapped genes in MSigDB gene sets, including BioCarta, KEGG, Reactome, and Gene Oncology (GO). The adjusted *P*-value (FDR) for gene set enrichment analysis is performed by the Benjamini-Hochberg procedure. We used the threshold of adjusted *P*-value 0.05 and the two minimum number of input genes overlapping with a tested gene. UCSC Genome Browser on Human Feb. 2009 (GRCh37/Hg19) Assembly was used to render the omics landscaping around the significant SNP regions.

## Results

### Characteristics of the study sample

To ensure retention of high-quality samples relevant to our research study, we followed strict protocol to retain only samples that met our quality thresholds (detailed descriptions of quality control procedures are provided in the “Methods” section). We retained 1683 ischemic stroke cases and 1738 stroke-free controls with a sex-stratified distribution of 1830 males and 1591 females after the application of stringent QC criteria. The demographic and risk factor characteristics by case-control status are described in Table 1. The mean age of the subjects with ischemic stroke was 61.2 (± 13.7) years, while the mean age of stroke-free control subjects was 59.5 (± 13.5) years (*P*-value = 0.0005). Consistent with previous observations, we demonstrated an abnormal waist-hip ratio as a strong risk factor for stroke (*P*-value < 0.0001). Cases were significantly more likely than controls to have a history of hypertension (95% vs. 63%) (*P*-value < 0.0001), diabetes (36% vs 14%) (*P*-value < 0.0001), dyslipidemia (73% vs. 61%) (*P*-value < 0.0001), and cardiac disease (13% vs. 6%) (*P*-value < 0.0001); we did not observe significant differences between cases and controls with respect to sex (*P*-value = 0.7062). We investigated clustering of potential ethnic differences in comparison with other 1000G populations using principal component analysis (PCA). The SIREN samples clustered together with 1000G African samples (Additional File 4: Fig. S1).

**Table 1:** Characteristics of the SIREN case-control samples after QC

Variable	Status/values	A: Controls (*N* = 1738)	B: Cases (*N* = 1683)	*P*-value comparing A and B
Baseline age (mean± SD)		59.5 ± 13.5	61.2 ± 13.7	0.0005
Sex (male/female)		924 (27.0%*)/814(23.8%)	906 (26.5%)/777 (22.7%)	0.7062
Hypertension (male/female)	No-risk	369 (10.8%)/274 (8%)	51 (1.5%)/32 (0.9%)	<0.0001
	Risk	554 (16.2%)/540 (15.8%)	851 (24.9%)/742 (21.7%)	
	Missing	1/0	4/3	
Diabetes (male/female)	No-risk	805 (23.5%)/691 (20.2%)	615 (18.0%)/458 (13.4%)	<0.0001
	Risk	118 (3.4%)/123 (3.6%)	289 (8.4%)/318 (9.3%)	
	Missing	1/0	2/1	
Dyslipidemia (male/female)	No-risk	374 (10.9%)/301 (8.8%)	252 (7.4%)/200 (5.8%)	<0.0001
	Risk	549 (16.0%)/513 (15.0%)	650 (19.0%)/575 (16.8%)	
	Missing	1/0	4/2	
Waist-to-hip ratio		0.921 ± 0.091	0.945 ± 0.078	<0.0001
Cardiac status (male/female)	No-risk	875 (25.6%)/763 (22.3%)	783 (22.9%)/668 (19.5%)	<0.0001
	Risk	46 (1.3%)/51 (1.5%)	117 (3.4%)/106 (3.1%)	
	Missing	3/0	6/3	
Ethnicity (male/female)	Akan	239/203	207/206	
	Yoruba	354/304	355/272	
	Hausa	149/135	138/126	
	GA/Adangbe	40/41	44/39	
	Ewe	39/24	34/24	
	Igbo	34/24	28/20	
	Other	69/74	95/83	
	Missing	0/7	5/7	
Toast status (male/female)	Large artery-atherosclerosis, embolus/thrombosis		255 (7.5%)/254 (7.4%)	
	Cardioembolism, high-risk/medium-risk		74 (2.2%)/53 (1.5%)	
	Small-vessel occlusion, lacune		343 (10%)/247 (7.2%)	
	Other determined etiology (dissection, vasculitis, cerebral venous sinus thrombosis, others)		3 (0.1%)/2 (0.1%)	
	Undetermined etiology (two or more causes identified, negative evaluation, incomplete evaluation)		231 (6.8%)/220 (6.4%)	

*% is calculated based on dividing by the total number of individuals in the study (*n* = 3421)

### Discovery genetic association analysis

**Fig. 1:**
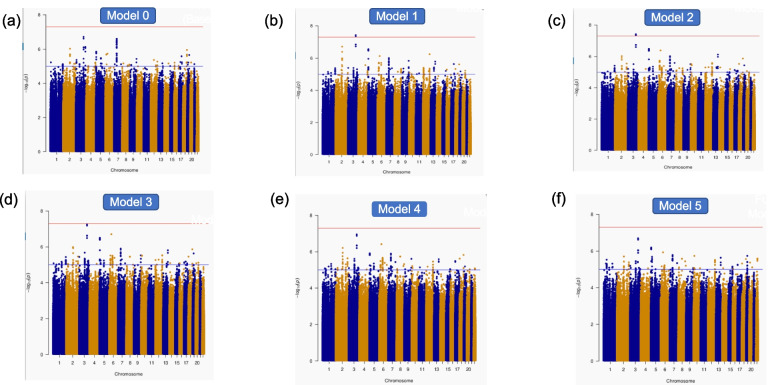
Manhattan plots. **a** The base model adjusted for sex, age, 10 PCs, and SNP as in model 0. **b** Hypertension is added to the base model 0. **c** Diabetes is added to the model 1. **d** Dyslipidemia is added to model 2. **e** Cardiac status is added to model 3. **f** Waist-to-hip ratio is added to model 4

**Fig. 2:**
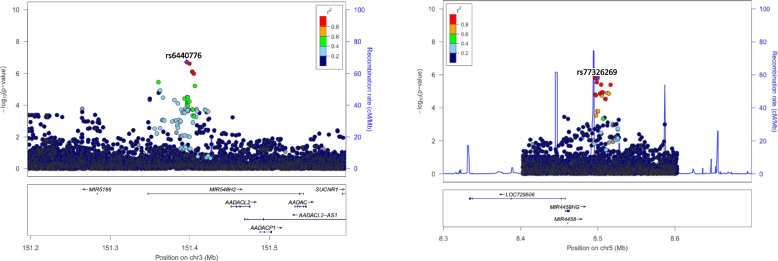
Locus zoom plots for SNPs rs6440776 (hg19: chr3:151396081 and hg38:151678293) and rs77326269 (hg19: chr5:8499398 and hg38:chr5:8499286) based on *P*-values using the base model

Manhattan plots for all six models are depicted in Fig. 1 starting with the primary/base model adjusted for sex, age, 10 PCs, and SNP. The base model was adjusted by adding one risk factor at a time hierarchically such as hypertension, diabetes, dyslipidemia, cardiac status, and waist-hip ratio. The quantile-quantile (QQ) plots are shown in Additional File 4: Fig. S2. We used the method proposed by Li and Ji based on spectral decomposition to estimate the effective number of SNPs (i.e., the number of independent SNPs) using 1,575,904 SNPs (MAF ≥ 0.01) (14, 15). We found that the number of independent SNPs are ~987,177 SNPs, which is close to 1M. We used a significance level of 5.06E−08 (= 0.05/987177) to correct for multiple testing. In Additional File 1: Table S1, we provide the ischemic stroke association with all SNPs in six models with *P*-value < 1.0E−6. Thirty-two [32] loci in chromosomes 2, 3, 5, 6, 7, 12, and 13 attained significance (*P*-value < 1.0E−6) in at least one of the six models. Note that there were only 7 independent SNPs. The goal was to show that these 7 SNPs had good linkage disequilibrium support, given in Additional File 1: Table S1. We observed genome-wide significant SNP associations near the *AADACL2* gene (distance ~50 kb) in chromosome 3 with the inclusion of hypertension to the base model [rs6440776, odds ratio (OR) of 0.73 with 95% CI: 0.66-0.82, *P*-value = 3.71E−08] (Table 2, Additional File 1: Table S1). Adding diabetes to the model in addition to hypertension, rs6440776 remained genome-wide significant. Furthermore, adding dyslipidemia to the model with hypertension and diabetes, the significance level was slightly below the genome-wide significance level for rs6440776 (rs6440776, OR 0.73 with 95% CI 0.66-0.82, *P*-value = 5.59E−08). Note that adding cardiac status and waist-hip ratio to the model, both SNPs remained significant with a significance level (*P*-value < 1.0E−06) (Table 2). Furthermore, a similar association pattern was observed in SNPs near the *MIR4458HG* gene (distance ~33 kb) in chromosome 5 with marginal significance (*P*-value < 1.0E−05) in all models (Table 2, Additional File 1: Table S1). Additional File 1: Table S2 contains the association results for any SNPs with a *P*-value < 1.0E−04. The Locus Zoom plots for SNPs in chromosomes 3 and 5 are shown in Fig. 2, and locus zoom plots for SNPs in chromosomes 2, 6, 7, 12, and 13 are shown in Additional File 1: Fig. S3. Note that the SNPs with suggestive significance in chromosome 2 were more than 85 kb from the closest gene *LINC01854*, and SNPs in chromosome 7 were more than 116kb to the closest *gene LINC01446*. In addition, we observed suggestive significance with SNPs in genes *CLIC5* (chromosome 6), *GALTN9* (chromosome 12), and closest gene *FAM155A* (chromosome 13) (*P*-value < 1.0E−5) in all five models ( Additional File 1: Table S1).

**Table 2:** Novel SNPs association with ischemic stroke*

Chr.	Position hg38	rsID	Gene(s)	Ref Allele	Alt Allele	HWE *P*-value	MAF	Model 0^#^ *P*-value	Model 1 *P*-value	Model 2 *P*-value	Model 3 *P*-value	Model 4 *P*-value	Model 5 *P*-value
3	151678293	rs6440776	*MIR5186-AADACL2*	C	T	0.13	0.37	1.93E−07	3.71E−08	3.81E−08	5.59E−08	1.11E−07	2.02E−07
5	8496166	rs57085808	*MIR4458HG*−*LINC02199*	G	C	0.37	0.12	1.49E−06	2.70E−07	3.22E−07	3.13E−07	1.22E−06	6.40E−07

*Only SNPs were included in the table if SNP has at least 5 SNPs in linkage disequilibrium (LD) within the genomic region and at least 3 of the models had *P*-value < 10^−6^

^#^Model 0: stroke status ~ sex + age + PCs1 … 10 + SNP; model 1: stroke status ~ sex + age + PCs1 … 10 + SNP + hypertension; model 2: stroke status ~ sex + age + PCs1 … 10 + SNP + hypertension + diabetes; model 3: stroke status ~ sex + age + PCs1 … 10 + SNP + hypertension + diabetes + dyslipidemia; model 4: stroke status ~ sex + age + PCs1 … 10 + SNP + hypertension + diabetes + dyslipidemia + cardiac disease status; model 5: stroke status ~ sex + age + PCs1 … 10 + SNP + hypertension + diabetes + dyslipidemia + cardiac disease status + waist-hip ratio

**Table 3:** Meta-analysis of SIREN and COMPASS studies for fixed effects (FE), conventional random effects (RE), alternate random effects (RE2), and binary effects (BE) models from METASOFT

Chr.	rsID	Position Hg38	Gene region hg38	Gene(s) hg38	Ref/Alt	*P*-value RE2	*P*-value BE	*I*^2^	SIREN *P*-value	COMPASS *P*-value	Effect size dir*
2	rs142655108	4036068	Intergenic	*LOC105373394-LINC01249*	C/A	7.16E−07	2.70E−07	80.22	4.63E−01	9.64E−08	+ +
3	rs6440776	151678293	Intergenic	*MIR5186-AADACL2*	C/T	8.05E−06	6.48E−07	94.82	1.95E−07	8.74E−01	− -
	rs184221467	153407501	Ncrna intronic	*LINC02006*	G/A	5.51E−08	1.44E−07	0	1.92E−02	7.79E−07	+ +
5	rs116123543	169439420	Intergenic	*SLIT3-SPDL1*	C/T	1.21E−06	2.96E−06	0	3.87E−02	8.04E−06	+ +
	rs268515	9478268	Intronic	*SEMA5A*	C/T	6.70E−07	1.49E−06	0	6.39E−02	2.60E−06	+ +
7	rs2194650	53526642	Intergenic	*POM121L12-LINC01446*	T/C	4.57E−06	8.63E−07	93.37	2.53E−07	2.92E−01	+ +
	rs184901586	66707028	Intronic	*RABGEF1*	A/C	1.21E−06	2.63E−06	0	2.63E−01	1.89E−06	− -
8	rs112455974	1624708	Intronic	*DLGAP2*	C/A	1.59E−06	9.26E−07	65.99	4.51E−01	3.79E−07	+ +
	rs11995798	77080739	Intergenic	*PEX2-LOC102724874*	G/C	9.40E−07	2.51E−06	0	1.27E−03	1.06E−04	+ +
9	rs12348429	136761594	Intronic	*LCN15*	G/A	3.19E−07	8.87E−07	0	6.45E−03	1.21E−05	+ +
	rs565295967	69860276	Intronic	*C9orf135*	C/T	1.81E−06	7.43E−07	71.41	9.65E−01	2.41E−07	− +
10	rs184882114	133387813	Intronic	*PAOX*	C/T	8.82E−07	1.74E−05	96.27	8.23E−04	5.00E−06	− +
	rs150576982	22779332	Intergenic	*PIP4K2A-ARMC3*	G/C	3.71E−07	9.87E−07	0	2.41E−02	4.22E−06	+ +
	rs74469072	51787504	Intronic	*PRKG1*	G/T	7.49E−07	6.62E−07	52.72	3.17E−01	3.46E−07	+ +
	rs145597261	5543158	Intergenic	*OLFM5P-OR52H1*	T/C	3.45E−07	9.75E−07	0	4.89E−03	1.74E−05	− -
	rs7107345	5556565	Intergenic	*OR52H1-OR52B6*	A/C	2.38E−07	6.51E−07	0	3.03E−03	1.69E−05	− -
12	rs192977447	119104946	Intronic	*SRRM4*	T/A	1.70E−06	5.86E−07	93.21	1.23E−01	1.77E−07	− +
	rs55931441	120977406	Intergenic	*HNF1A-AS1-HNF1A*	G/A	2.82E−07	1.20E−07	70.68	5.99E−01	4.58E−08	+ +
14	rs10498430	51476243	ncRNA intronic	*FRMD6-AS2*	C/T	5.85E−07	1.60E−06	0	4.90E−03	2.85E−05	+ +
16	rs114318459	81767653	Intergenic	*CMIP-PLCG2*	T/C	1.12E−06	2.65E−06	0	4.50E−02	6.31E−06	− -
19	rs12982680	34661176	ncRNA intronic	*SCGB1B2P*	G/A	8.43E−07	2.25E−06	0	1.51E−02	1.50E−05	+ +
	rs187158875	39518254	Intronic	*SELENOV*	G/A	7.03E−07	1.46E−06	0	4.74E−02	2.97E−06	+ +
22	rs116127899	47568929	Intergenic	*LINC01644-LINC00898*	G/A	7.15E−07	1.51E−06	0	1.67E−01	1.46E−06	+ +

*Effect size direction order: SIREN, COMPASS; We used the baseline model from SIREN for meta-analysis: Stroke Status ~ Sex + Age + PCs1…10 + SNP

### Transferability analysis

Due to lack of a replication sample of indigenous Africans, we investigated the transferability of our findings in COMPASS (African-American meta-analysis) and MEGASTROKE (European Ancestry Meta-Analysis). Additional File 1: Table S3a shows the statistical significance in COMPASS (column BB provides the *P*-values in COMPASS) and MEGASTROKE (column BI for *P*-values in MEGASTROKE) for top SIREN hits corresponding to Additional File 1: Table S1 with *P*-value < 1.0E−06. Additional File 1: Table S3b presents significance levels in COMPASS and MEGASTROKE based on SIREN *P*-value < 1.0E−04 corresponding to Additional File 1: Table S2. Note that there were only two SNPs rs116683655 and rs76250200 within gene *ISPD* with *P*-value < 1.0E−04 in SIREN were marginally significant with *P*-values 3.98E−03 and 5.57E−03 in COMPASS, respectively. The lowest *P*-value in MEGASTROKE was 8.38E−03 corresponding to SIREN with *P*-values < 1.0E−04 for the SNP rs7239115 in chromosome 18 within gene region *LINC01898*-*LOC339298*. Conversely, we also investigated the transferability status of variants previously associated with stroke in COMPASS and MEGASTROKE in SIREN (Additional File 1: Table S4a and S4b). In Additional File 1: Table S4a, S4b, and S13a-d, we have identified and listed out specific SNPs associated with stroke risk among African-Americans and Europeans as identified in the COMPASS and MEGASTROKE studies respectively. SNPs labeled as multi-ancestry were also identified. We observed a nominal association with multiple SNPs in COMPASS including rs116262092 (*P*-value = 0.02) and rs147867382 (*P*-value = 0.02) in the *RUNX1* gene in chromosome 21, rs184221467 (*P*-value = 0.02) near the *AK092619* gene in chromosome 3, and rs115670077 (*P*-value = 0.01) between the *RFTN2*-*MARS2* gene in the SIREN cohort with a similar direction of effect as in the COMPASS. Additional analysis comparing the effect sizes of the variants across the COMPASS and SIREN cohorts demonstrated similar effect sizes and direction of effect in most of the loci.

We further investigated the transferability status of variants previously associated with stroke subtypes in COMPASS and MEGASTROKE in SIREN. We replicated the top significant SNPs associations in COMPASS and MEGASTROKE in the SIREN for large artery disease (cases = 509 vs. controls = 1738), small vessel occlusion (cases = 590 vs. controls = 1738), and undetermined etiology (cases = 451 vs. controls = 1738). None of the top loci in COMPASS or MEGASTROKE were significant with Bonferroni correction for any of the subtypes. The results for subtypes corresponding to COMPASS and MEGASTROKE are provided in Additional File 1: Table S5a and S5b, respectively, and showed marginally significant results in subtypes with *P*-value < 0.05 in SIREN. The effect sizes are in the same directions as in COMPASS and MEGASTROKE except for SNP rs113025543 (*FAR*) which is a protective factor in COMPASS but a risk factor in SIREN for small-vessel disease and SNP rs11867415 (*PRPF8*) which is a risk factor in MEGASTROKE but protective in SIREN for small-vessel disease (Additional File 1: Table S5c contains the summary of the marginally significant results of the subtypes in SIREN).

### African ancestry meta-analysis

Additional File 1: Table S6 contains the results from METASOFT for *P*-values < 1.0E−04 corresponding to the RE2 model. There were 14,053,108 SNPs common to both SIREN and COMPASS. Table 3 provides a summary of the METASOFT results with *P*-values less than 1.0E−06 for Han and Eskin’s random effects model (RE2) and the binary effects model (BE) for meta-analysis models, heterogeneity value *I*^2^, and corresponding SIREN and COMPASS *P*-values and their effect size directions. There were 15 SNPs in Han and Eskin’s random effects model (RE2) and 13 SNPs in the binary effects model (BE) with *P*-value < 1.0E−06. COMPASS SNPs drove most of the SNP significance in the RE2 model. However, SIREN SNPs were significant for BE model with *I*^2^ greater than or equal to 0.90 with *P*-value < 1.0E−06. Note that rs6440776 in the intergenic region of *MIR5186-AADACL2* in chromosome 3 and rs2194650 in *POM121L12-LINC01446* were also significant with a *P*-value less than 1.0E−06 in the BE model corresponding to SIREN *P*-value < 1.0E−06. Moreover, the direction of effect between associations of the loci with ischemic stroke in both SIREN and COMPASS studies were similar for 2504 SNPs out of 3111 in Additional File 1: Table S6 and SIREN vs. COMPASS effect size plot in Additional File 4: Fig. S4.

### Transethnic meta-analysis

Transethnic meta-analysis was performed in MANTRA using SIREN, COMPASS and, MEGASTROKE studies. There were 6,092,926 SNPs common to all three studies. The MANTRA results with log10 (Bayes factor) > 4 are included in Additional File 1: Table S7. A summary of the MANTRA results is given in Table 4 containing log_10_ (Bayes factor) ≥10.0. The significance of the all SNPs in Table 4 was mainly driven by MEGASTROKE SNP’s *P*-values and their effect sizes. Note that MEGASTROKE was the largest study among all three studies, with a sample size of 446,696, while COMPASS had 22,051 individuals compared with SIREN with 3434 individuals. It is not uncommon for a meta-analysis to be heavily dominated by a single largest study [35, 36]. We observed that allele frequency distributions in MEGASTROKE were different compared to COMPASS and SIREN (see Additional File 4: Fig. S5). COMPASS and SIREN allele frequency distributions were similar (see Additional File 4: Fig. S5). There were 231 SNPs with log_10_ (Bayes factor) ≥ 6.0, and most of the SNPs were significant in MEGASTROKE. SIREN study-driven MANTRA results are given in Table 5 with Bayes factor of at least 4.0 with posterior probability of 1 and SIREN *P*-value < 1.0E−04. Both SNPs rs6440776 and rs2410883 in *MIR5186-AADACL2* in chromosome 3 had Bayes factor greater than 5 with effects in the same direction in all three studies. The SNPs in chromosomes 7, 18, and 20 had Bayes factor greater than 4.0 with a posterior probability of 1 corresponding to SIREN *P*-values < 1.0E−04.

**Table 4:** Results from MANTRA using SIREN, COMPASS, and MEGASTROKE studies with Log_10_ (Bayes factor)≥10

Chr.	Pos (Hg38)	rsSNP	Gene(s) hg38	Gene regions hg38	Ref/Alt	log10 Bayes factor	Posterior probability	Total samples	SIREN *P*-value	COMPASS *P*-value	MEGASTROKE *P*-value	Effect size dir*
4	110755885	rs2634074	*PITX2-MIR297*	Intergenic	A/T	11.32	0.463	471334	0.797	0.383	5.91E−15	+−−
	110764459	rs2466455	*PITX2-MIR297*	Intergenic	C/T	11.36	0.478	472086	0.920	0.445	8.87E−15	−++
	110767596	rs2723334	*PITX2-MIR297*	Intergenic	C/T	10.89	0.629	472166	0.800	0.511	1.49E−14	+−−
	110775495	rs6847935	*PITX2-MIR297*	Intergenic	T/A	11.20	0.826	470846	0.093	0.288	1.80E−14	−++
	110776412	rs1906616	*PITX2-MIR297*	Intergenic	G/A	10.45	0.83	472040	0.118	0.311	3.98E−14	−++
	110776952	rs6837901	*PITX2-MIR297*	Intergenic	C/T	11.02	0.828	472049	0.118	0.342	1.54E−14	−++
	110778529	rs67249485	*PITX2-MIR297*	Intergenic	T/A	10.93	0.824	472063	0.118	0.299	1.65E−14	−++
	110778675	rs6820568	*PITX2-MIR297*	Intergenic	T/C	10.93	0.811	472056	0.143	0.378	1.83E−14	−++
	110780642	rs1906615	*PITX2-MIR297*	Intergenic	T/G	10.57	0.635	472077	0.320	0.375	4.41E−14	−++
	110782924	rs2129983	*PITX2-MIR297*	Intergenic	G/A	11.28	0.305	471995	0.797	0.176	2.36E−14	−++
	110782987	rs2129982	*PITX2-MIR297*	Intergenic	G/A	10.78	0.867	472167	0.073	0.287	5.86E−14	−++
	110783380	rs6854111	*PITX2-MIR297*	Intergenic	T/A	11.12	0.573	471394	0.246	0.214	1.99E−14	−++
	110784139	rs12639654	*PITX2-MIR297*	Intergenic	T/C	10.02	0.129	469158	0.760	0.400	2.01E−12	−++
	110791276	rs2129977	*PITX2-MIR297*	Intergenic	A/G	11.40	0.398	472105	0.393	0.136	2.65E−14	−++
	110796911	rs6843082	*PITX2-MIR297*	Intergenic	G/A	10.28	0.261	471736	0.573	0.122	6.73E−13	−++
7	19009765	rs2107595	*HDAC9-TWIST1*	Intergenic	A/G	10.40	0.072	472122	0.137	0.053	2.33E−11	+++
12	111395984	rs10774624	*FAM109A-SH2B3*	Intergenic	G/A	11.93	0.139	469904	0.430	0.224	7.66E−14	−++
	111466567	rs4766578	*ATXN2*	Intronic	T/A	10.81	0.143	469940	0.423	0.185	1.36E−12	−++
	111494996	rs7137828	*ATXN2*	Intronic	C/T	11.00	0.141	469942	0.422	0.173	6.61E−13	−++
	111569952	rs653178	*ATXN2*	Intronic	C/T	11.58	0.141	469939	0.410	0.228	1.59E−13	−++
	111634620	rs11065987	*ATXN2-AS-BRAP*	Intergenic	G/A	10.12	0.152	469938	0.467	0.747	5.81E−13	−++
	112049014	rs17696736	*NAA25*	Intronic	G/A	10.32	0.176	469948	0.498	0.518	2.16E−12	−++

*Effect size direction order: SIREN, COMPASS, and MEGASTROKE; We used the baseline model from SIREN for MANTRA analysis: Stroke Status ~ Sex + Age + PCs1…10 + SNP

**Table 5:** SIREN study driven MANTRA results with Log_10_ (Bayes factor)≥4.0^*^

Chr.	Position (hg38)	rsID	Gene(s) hg38	Gene region hg38	Ref/Alt	Log_10_ Bayes factor	Posterior probability	Total samples	SIREN *P*-value	COMPASS *P*-value	MEGASTROKE *P*-value	Effect size dir^#^
3	151678293	rs6440776	*MIR5186-AADACL2*	Intergenic	T/C	5.102	1	472150	1.94E−07	0.873	0.468	−−−
3	151681716	rs2410883	*MIR5186-AADACL2*	Intergenic	G/A	5.039	1	472150	2.46E−07	0.688	0.493	−−−
7	53526642	rs2194650	*POM121L12-LINC01446*	Intergenic	T/C	4.398	1	472156	2.52E−07	0.292	0.604	++−
7	53527922	rs4541851	*POM121L12-LINC01446*	Intergenic	G/A	4.733	1	472156	2.89E−07	0.308	0.493	++−
7	53532401	rs10230418	*POM121L12-LINC01446*	Intergenic	G/A	4.393	1	472144	3.38E−07	0.279	0.630	++−
7	53520258	rs10250977	*POM121L12-LINC01446*	Intergenic	T/C	4.456	1	472162	3.42E−07	0.297	0.626	++−
7	53515752	rs11238270	*POM121L12-LINC01446*	Intergenic	C/T	4.270	1	472162	3.73E−07	0.272	0.639	++−
7	53516754	rs4947814	*POM121L12-LINC01446*	Intergenic	C/A	4.255	1	472163	4.08E−07	0.164	0.718	++−
7	53534088	rs1559587	*POM121L12-LINC01446*	Intergenic	A/T	4.452	1	472156	5.35E−07	0.286	0.619	++−
18	75791508	rs7239115	*LINC01898-LOC339298*	Intergenic	C/T	5.780	1	472023	1.10E−06	0.126	0.008	−++
18	75787931	rs898487	*LINC01898-LOC339298*	Intergenic	G/A	4.594	1	472143	2.06E−06	0.128	0.108	−++
18	75788278	rs7228334	*LINC01898-LOC339298*	Intergenic	C/T	5.491	1	472131	2.50E−06	0.128	0.012	−++
20	19535610	rs6035372	*SLC24A3*	Intronic	G/A	4.253	1	472167	2.28E−05	0.102	0.046	+−−

*Mantra results with Bayes Factor ≥ 4 for significant loci in SIREN with *P*-value<1.0E-04

^**#**^Effect size direction order: SIREN, COMPASS, and MEGASTROKE; We used the baseline model from SIREN for MANTRA analysis: Stroke Status ~ Sex + Age + PCs1…10 + SNP

### Fine-mapping

**Fig. 3:**
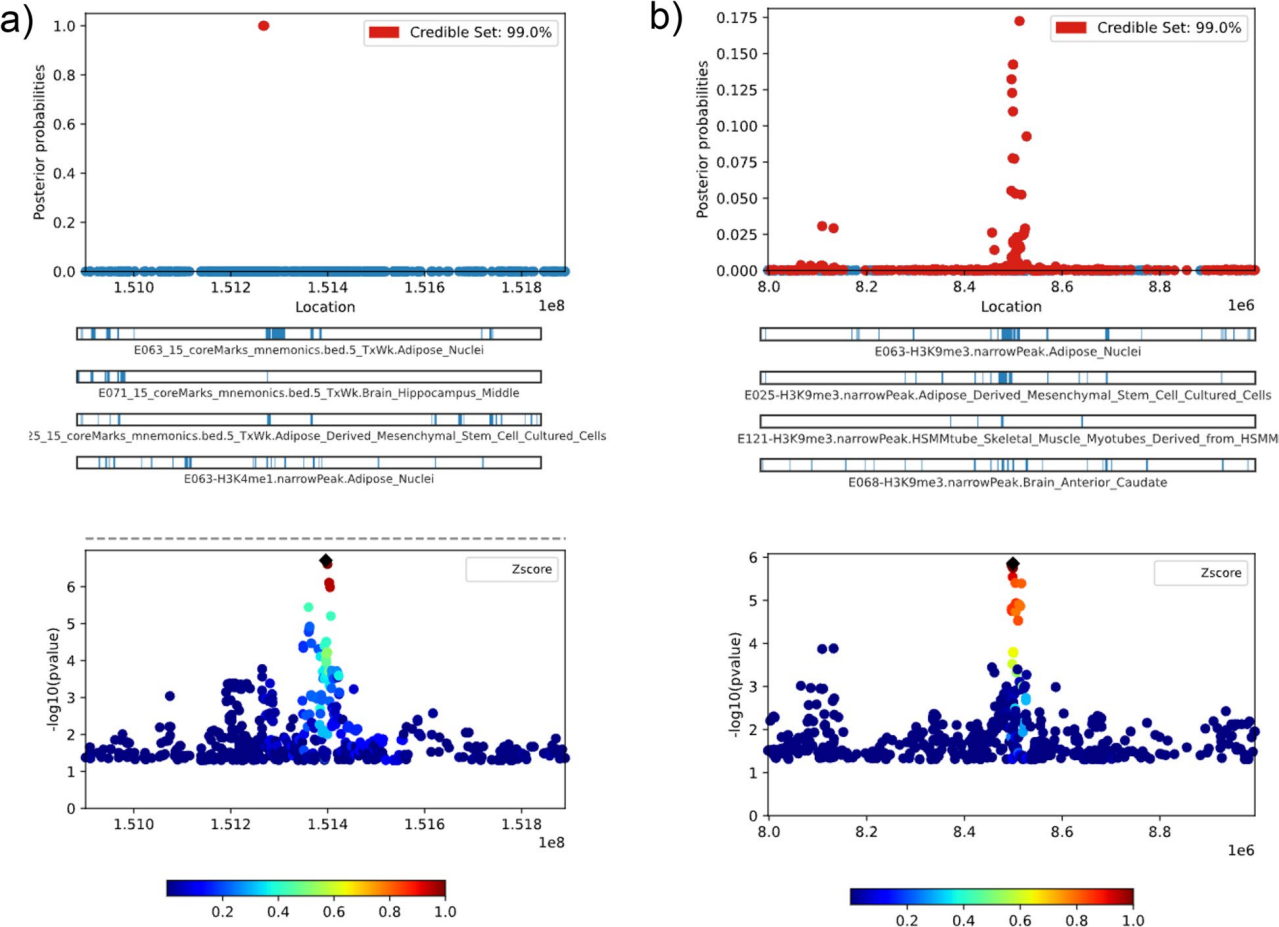
**a** Fine-mapping of AADACL2 gene region. **b** MIR4458HG gene region. Panel 1 depicts a scatterplot of location versus posterior probabilities with a 99% credible interval; panel 2 provides functional annotation tracks

Before performing fine-mapping, localized zoom plots in Fig. 2 were consulted for both regional association landscape and linkage disequilibrium with the lead variant in the region of interest. Fine-mapping regions were initially identified using a genomic base-pair window size of 500 kb on both 5′ and 3′ ends of the significant hits near *AADACL2* and *MIR4458HG* genes based on the hg19 coordinate system. Fine-mapping in chromosome 3 indicated 2 variants out of the 627 variants considered were potentially causal (rs7611359, position: 151266619, posterior probability = 1.0 with 99% credible interval; and rs9815407, position: 151269245, posterior probability = 1.0 with 99% credible interval) (Fig. 3a). Similarly, fine mapping in chromosome 5 indicated 4 out of the 568 variants considered were potentially causal (rs341875, position: 8512751, posterior probability = 0.17 with 99% credible interval; rs77326269, position: 8499398, posterior probability = 0.14 with 99% credible interval; rs73740017, position: 8499591, posterior probability = 0.14 with 99% credible interval; and rs57085808, position: 8496279, posterior probability = 0.13 with 99% credible interval) (Fig. 3b). To select the top five 10 annotation sets for each region, we employed the suggested pipeline outlined in the PAINTOR software GitHub repository. Additional File 1: Table S8a and S9a capture the marginal significance estimates for each annotation and the overall likelihood ratio test (LRT) estimates, which were used to select the top 10 annotations of interest.

### Gene sets enrichment analysis

To determine gene expression profile tissue/cell type specificity for our genes of interest, we used a gene lookup mechanism in GTExPortal V8 (https://www.gtexportal.org). The gene expression analysis in GTExPortal V8 for *MIR4458HG* and *AADACL2* genes is shown in Additional File 4: Fig. S6a and S6b. The highest expression was observed in brain-cerebellar hemisphere and brain-cerebellum for the *MIR4458HG* gene. To further understand any functional implications of significant single variant association analysis, we performed functional annotation mapping (FUMA) GWAS module SNP2GENE. We used any SNPs in any model with a *P*-value < 1.0E−5 for SNP2GENE analysis. MAGMA tissue-specific expression analysis results of SNP2GENE module are given in Additional File 1: Table S10a. Tissue-specific expression analysis with *P*-value < 0.05 was observed in thyroid, brain cerebellar hemisphere, and brain cerebellum tissues. In addition, we performed GENE2FUNC using a compilation of 191 genes that were aggregated from ANNOVAR gene assignment report for SNPs in Additional File 1: Table S2 and genes that showcased chromatin and eQTL interactions based on SNP2GENE results. The 143 genes with recognized unique Ensembl ID were used in annotation and mapping. In specific tissue analysis, FUMA GENE2FUNC differentially expressed genes were either upregulated or downregulated. Enrichment for upregulated gene differential expression in the brain was observed in brain spinal cord cervical C-1 (*p*_adj_ = 0.043) and downregulated in brain frontal cortex BA9 (*p*_adj_ = 0.032) along with brain cortex (*p*_adj_ = 0.090). The details regarding upregulated and downregulated are provided in Additional File 4: Fig. S6a and S6b and Additional File 1: Table S10b. We also observed two-sided significant regulation of genes in specific tissues, namely brain frontal cortex BA9 (*p*_adj_ = 0.023).

### Genomic landscaping for genes AADACL2 and MIR4458HG

**Fig. 4:**
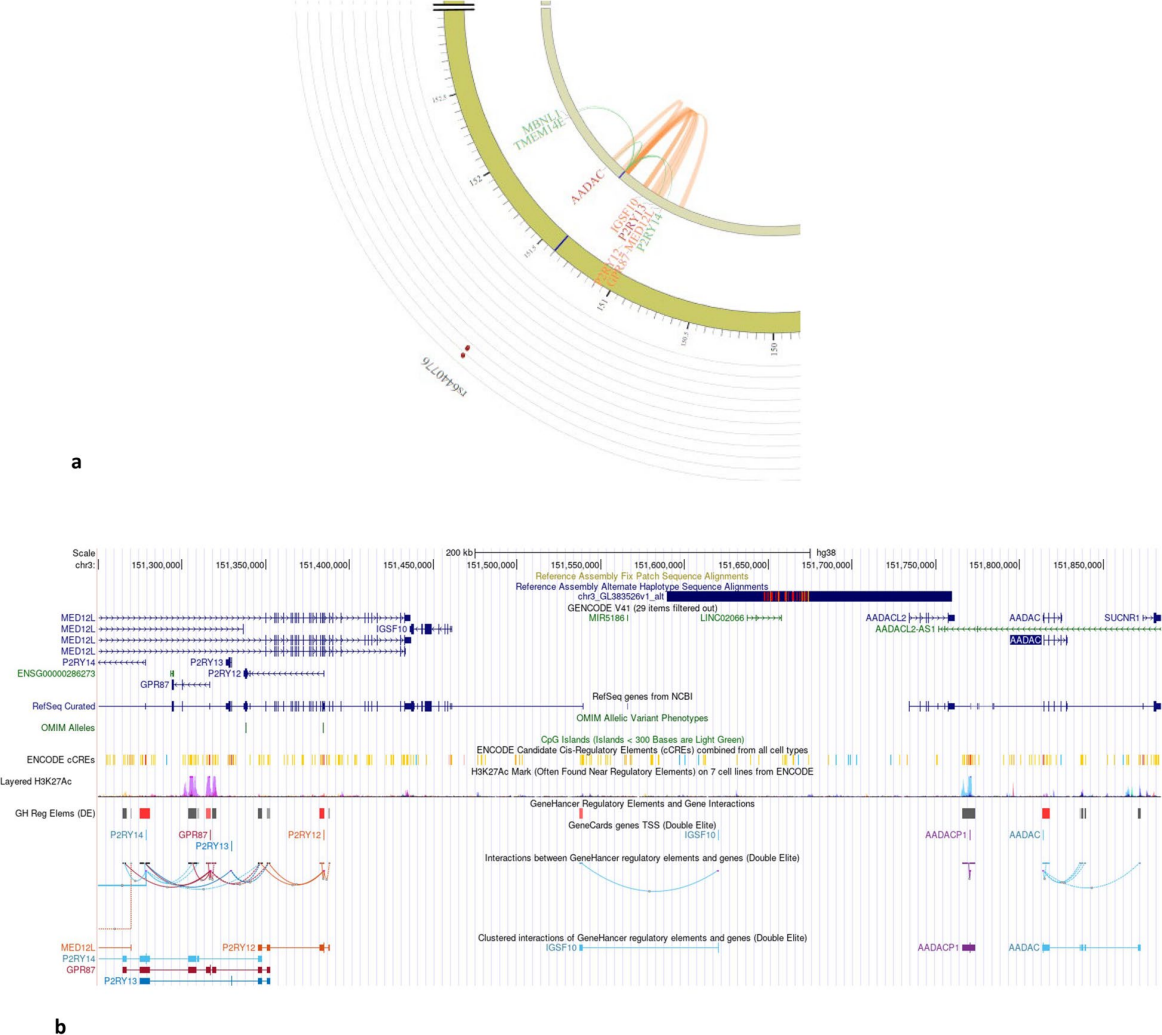
**a** Circos plot showcasing chromatin interaction (orange arcs) and eQTL interactions (green arcs) originating from SNP rs6440776 (AADAC gene region). **b** Genomic landscape for AADACL2 illustrating CpG islands, enhancer/promoter presence, histone modification sites, and regulatory interaction activity from UCSC browser

**Fig. 5:**
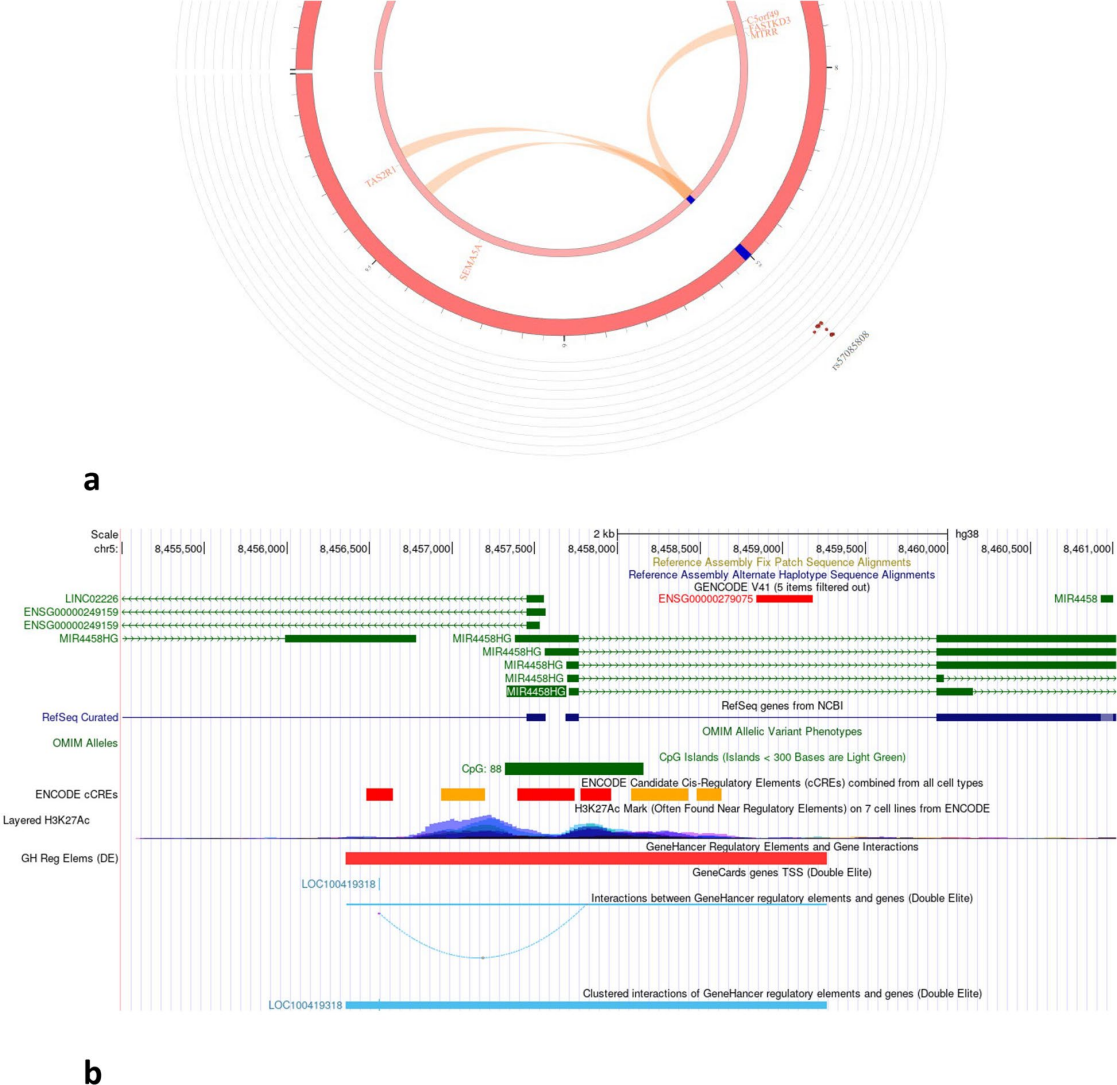
**a** Circos plot showcasing chromatin interaction (orange arcs) and eQTL interactions (green arcs) originating from SNP rs57085803 (MIR4458HG gene region). **b** Genomic landscape for MIR4458HG illustrating CpG islands, enhancer/promoter presence, histone modification sites, and regulatory interaction activity from UCSC browser

Given the dense distribution of variants in and around the lead significant SNP, localized genomic visualization models, Figs. 4 and 5, were rendered to investigate the (1) presence of methylation hotspots in the form of CpG islands/shores, (2) observance of enhancer and promoter activity reported by GeneHancer, and (3) interaction between GeneHancer regulatory elements and neighboring genes. Furthermore, brain DNA methylation profile was also investigated in and around the region of significant SNPs, and the same is showcased as independent tracks in the rendered regions (a) genome-wide methylation (MeDIP-seq and MRE-seq) landscape, (b) histone H3 lysine 4 trimethylation (H3K4me3), and (c) gene expression (RNA-seq and RNA-seq (SMART)) profiles. Figure 4a illustrates the chromatin interaction link between significant regions proximate to *AADACL2* gene and nearby *IGSF10* gene using SNP2GENE function in FUMA. Additional File 1: Table S11 articulates the significant intra-chromosomal chromatin interaction and strength of SNP-gene-tissue eQTL mapping for genome-wide significant SNP regions along with novel SNPs near *AADACL2*. Based on the GWAS significance statistics for SNPs in that region, *P2RY13 and P2RY14* are potential eQTLs with significant mapping interaction with the novel SNPs near *AADACL2*. UCSC Genome Browser on Human Hg38 build was used to render the omics landscaping around the significant SNP regions. *AADCL2* omics landscape in Fig. 4b reports minimal promoter and enhancer presence. Interestingly, 5 clustered interactions of gene enhancer regulatory elements and the *AADAC* gene, which is located only 56 kb downstream of *AADACL2*, were observed around the region of the *AADACL2* gene. Figure 5a depicts the chromatin interaction of rs57085808 with nearby genes. As depicted in Fig. 5b, the presence of methylation hotspot, CpG Island, at the 5′ end of *MIR4458HG* demonstrated a high level of H3K27Ac epigenetic modification signal. H3K27Ac histone mark is known to be a strong marker of active promoter and enhancer activity that is strongly associated with the transcription factor binding mechanism and gene expression profile. Histone mark’s activity is further validated by the presence of a cluster of strong active promoter regions (red bands) along with transcriptional transition and elongation (green bands) hotspots, thereby offering some potential interaction between DNA methylation and histone modifications around the region of *MIR44*58HG gene. Additional File 1: Table S12 contains the significant intra-SNP-gene-tissue eQTL mapping for gene *MIR4458HG*.

### Genome geography discrepancies

Although there is healthy validation and verification of sequence similarity between multiple gene transcripts for a certain genomic region, genomic annotations are yet to reach robust levels of certainty and stability across evolving versions of human reference genomes. Although we employed TOPMed imputation reference panel with human genome Hg38, much of our replication cohorts like COMPASS and MEGASTROKE reported their variants based on Hg37. To accommodate potential inconsistences between these two different versions of the human genome, we presented variant annotations in our additional file datasets for both Hg19- and Hg38-based coordinate systems. At a glance, the genome versioning challenge also helped us unravel few issues with annotating SNPs for assigning HUGO approved gene names, genomic functions, and SNP annotations. One of our top-hit variant rs6440776 was reported on chr3:151678293 based on Hg38 genome assembly and on chr3:151396081 based on Hg37 assembly. Based on the version of the assembly used, SNP rs6440776 was mapped to intergenic regions between genes *MIR5186-AADACL2* based on Hg38 and mapped to ncRNA intronic region of gene *MIR548H2* based on hg19. Also, based on the version of the dbSNP data repository used to drive the SNP annotations, the same variant on Chr2 at position 129359443 (Hg38) with mapped position 130117016 (Hg19) was assigned registered dbSNP name rs111452560 and rs116332314 between different data releases of dbSNP database.

## Discussion

In this first genome-wide association study of ischemic stroke among indigenous Africans, we observed genome-wide significant SNPs associations (rs6440776 and rs2410883) near the *AADACL2* gene in chromosome 3, after adjusting for hypertension, diabetes, and dyslipidemia in the base model as covariates. Five SNPs (rs57085808, rs57033994, rs143745837, rs77326269 and rs73740017) near the miRNA (*MIR4458HG*) gene in chromosome 5 were also associated with ischemic stroke with suggestive significance (*P*-value < 1.0E−6)). The loci near *AADACL2* and *MIR4458HG* genes are novel and protective. The region near gene AADACL2 remained marginally significant following African ancestry meta-analysis and fine mapping. The functional and clinical relevance of the identified risk loci is further supported by eQTL and chromatin interaction data. The observed protectiveness of these loci against stroke has promising implications for ancestry-specific risk stratification and the search for drug targets that can enhance the primary or secondary prevention of stroke (please see Additional File 3: Additional Discussion (additional discussion point a and additional discussion point b) on other marginally significant genetic variants).

The arylacetamide deacetylase like 2 (*AADACL2*) gene is a protein coding gene that is strongly expressed in the skin, an organ that shares embryological origins with the nervous system. The gene is implicated in epidermal barrier function [37] and has demonstrated previous associations with multiple phenotypes including idiopathic dilated cardiomyopathy [38]. Loci near *AADACL2* in the present study demonstrate protection against ischemic stroke with top SNPs: rs6440776 with OR 0.74 (0.66–0.82) and *P*-value = 3.71E−08 and rs2410883 with OR 0.74 (0.66–0.82) and *P*-value = 4.38E−08 when hypertension was included in the model.

Fine-mapping of the significant genomic regions near the *AADACL2* gene in chromosome 3 yielded two potentially causal variants rs7611359 and rs9815407 with a posterior probability of 1.0. Gene expression profiling results for the *AADACL2* gene using GTEx v8 yielded maximum expression in the skin while genomic landscaping yielded minimal enhancer, histone modification, and regulatory interaction activity. In addition, 5 clustered interactions of gene enhancer regulatory elements and the *AADAC* gene located 56 kb downstream of *AADACL2* were observed around the region of the *AADACL2* gene. The significant histone modification and regulatory activity of the novel loci near the *AADACL2* gene plausibly explain the protection against ischemic stroke demonstrated in this study. Potential interactions involving the discovery novel loci near *AADACL2* in this study and other genes, particularly in proximity within the chromosome 3, may also explain the protective function of the novel loci in relation to ischemic stroke. Chromatin interaction mapping of regions proximate to the *AADACL2* gene demonstrated significant intra-chromosomal chromatin interaction with the *IGSF10* (immunoglobulin superfamily, member 10) gene with relevant immune regulatory functions [39].

The *MIR4458HG* gene is an intergenic non-coding miRNA gene with multiple tissue expression in the brain, arteries, and other tissues [40, 41] as well as metabolite level and heart rate in heart failure with reduced ejection fraction [42]. The *MIR4458HG* gene was previously associated with coronary artery calcification in a GWAS study among type 2 diabetes in African-American/Afro-Caribbean subjects [43]. In this study, SNPs near the *MIR4458HG* gene locus demonstrated protection against ischemic stroke with ORs < 1 at suggestive significance levels.

Fine-mapping of the significant genomic regions near the *MIR4458HG* gene in chromosome 5 yielded 4 variants considered potentially causal, top of which was rs341875 with a posterior probability of 0.17. Gene expression analysis was undertaken for the *MIR4458HG* gene in GTExPortal V8 in both general and specific tissues. This demonstrated the highest expression in the brain cerebellar hemisphere, cerebellum, and thyroid as well as artery tibial and coronary arteries. Functional annotation mapping (FUMA) expression analysis in MAGMA demonstrated differential gene expression in the brain spinal cord cervical C1 and brain frontal cortex BA9. Genomic landscaping for *MIR4458HG* yielded methylation signals, strong enhancer/promoter activity, histone modification sites, and regulatory interaction activity with the high level of H3K27Ac epigenetic modification signaling. These findings demonstrate epigenetic interactions including DNA methylation and histone modifications around the *MIR4458HG* gene and thus suggest regulatory activity in the variants near the *MIR4458HG* gene as a plausible mechanism for the protective effect on ischemic stroke and the consequent potential of the region containing targets for drug development for primary or secondary prevention of stroke [11].

A recent cell culture study demonstrated that miR-4458 negatively modulated cardiac hypertrophy, a known intermediate phenotype, and an independent risk factor for ischemic stroke, by activating mitochondrial transcription factor A (TFAM), a well-recognized myocardial protective protein. Indeed, miR-4458 facilitated TFAM expression in cardiomyocytes to inhibit cardiac hypertrophy [44]. Several other micro-RNA genes have also demonstrated protection against ischemic stroke such as miR-375 [45], miR-195 [46], miR-221 [47], miR-338 [48], and exhibiting protection against ischemic stroke via multiple mechanisms. Moreover, microRNAs constitute an emerging and promising category of biomolecules with the promise of enhancing risk prediction, diagnosis, prognosis, and treatment of ischemic stroke and the subtypes [49–51].

### Clinical implications of functional expressions and interaction analysis

Expression quantitative trait loci (eQTL) mapping and chromatin interaction analysis in FUMA demonstrate interaction of variants with either genomic or suggestive significance with other multiple variants with significant expression in vascular or brain tissue and association with cerebrovascular disease phenotypes, other brain disorders, or vascular diseases (Additional File 1: Table S10 and S11). For instance, novel loci near the *AADACL2* gene yielded potential eQTLs including *AADAC*, *MBNL1*, *TMEM14E*, *P2RY13*, and *P2RY14* genes with *P2RY13* and *P2RY14* demonstrating significant mapping interaction. The purinergic receptor (*P2Y13*) plays a major role in HDL metabolism by facilitating reverse cholesterol transport and promoting the inhibition of atherosclerosis progression) [52–54]. Thus, it appears that the protectiveness of the novel locus near *AADACL2* against stroke may be associated with its epistatic interaction with the *P2RY13* gene. Systems genetics analysis has also defined the importance of transmembrane protein 43 (TMEM43) in cardiac- and metabolic-related pathways, **s**uggesting that cardiovascular disease-relevant risk factors may also increase risk of metabolic and neurodegenerative diseases via *TMEM43*-mediated pathways [55]. Broad cellular functions and diseases including arrhythmogenic right ventricular cardiomyopathy (ARVC5) have been associated with transmembrane protein43 (TMEM43).

Taken together, the findings in this study demonstrate emerging differential roles for regulatory miRNA, intergenic non-coding DNA, and intronic non-coding RNA in the pathobiology of ischemic stroke. The protectiveness of some genetic loci related to miRNAs, which are largely regulatory, suggests the possible occurrence of downstream biomolecules and processes in dysregulated pathways and networks, which require further exploration and characterization. Indeed, multiple loci which demonstrate significant interaction with our key discovery variants (with regulatory function) through FUMA have shown expression in brain, vascular, cardiac, and neuronal tissue apart from direct association with different subtypes of cerebrovascular disorders. These have implications for novel fluid biomarkers for stroke, drug development, and repurposing, multi-omics analysis including genome-wide miRNA analyses, and generation of polygenic risk score (PRS) that will likely be more accurate for African populations [56–58].

### Comparison with existing stroke GWAS

Replication is a critical part of the process of studying genome-wide association studies, while the concept of transferability is used when the replication cohort is drawn from a different population other than the discovery sample [59, 60] (please see additional discussion point c in Additional File 3: Additional Discussion). Findings from the SIREN discovery analysis demonstrated poor transferability in the COMPASS meta-analyses among African-Americans [8, 9] and vice versa possibly because of genetic admixture in the African-Americans. However, the similarity of direction of effect between the associations of the loci with ischemic stroke in both SIREN and COMPASS studies strengthens the biological validity of the association of these loci with ischemic stroke (Additional File 4: Fig. S4) [13]. Similarly, the findings from the MEGASTROKE meta-analysis [10] showed non-transferability in both SIREN and COMPASS GWAS analyses. The MEGASTROKE GWAS was in a predominantly European ancestry population with only 4.0% African ancestry (African-Americans) which is slightly more than the 3.7% African ancestry in GIGASTROKE [19]. Differences in the ancestral backgrounds of the SIREN and MEGASTROKE cohorts and the dominance of small vessel disease stroke subtype among blacks compared to Caucasians are plausible reasons for this non-transferability. A recent high-depth study of African genomes identified more than 3 million previously undescribed genetic variants [18]. This observation underscores the uniqueness of the genetic architecture of indigenous African populations with variants which may not be present in other populations. This has implication for the non-transferability in this study and other African studies (DM, glaucoma and lipid traits) [13, 61, 62] (please see additional discussion points d and e in Additional file 3: Additional Discussion). The existence of such ancestry-specific variants has implications for the development of polygenic risk scores (PRS) of higher accuracy in the stratification of individuals based on disease risks. This therefore strengthens the argument for ancestry or region-specific PRS.

### Strengths, limitations, and future direction

Our study has a major strength in being the first stroke GWAS in an indigenous African population with novel functional and clinical implications. The key limitations are the absence of a suitable independent replication cohort of indigenous African ancestry and the non-availability of databases enriched with African ancestry information for in silico functional analysis. These could have limited the full understanding of the functional implications of our discoveries. This limitation is particularly common to pioneering GWAS studies of African ancestry individuals such as the recent GWAS of rheumatic heart disease [63]. The current study was also not sufficiently powered for stroke sub type-specific analysis to identify ischemic stroke sub type-specific risk loci. We found marginally significant transferability upon investigation of variants associated with ischemic stroke subtypes due to small vessel disease and large artery atherosclerosis. Future larger stroke GWAS studies are required to accurately dissect the genetic and pathological heterogeneity between ischemic stroke subtypes among indigenous Africans. We investigated the functional relevance of the identified risk loci using bioinformatic analyses that we plan to confirm via in vitro and in vivo studies in the near future.

## Conclusions

In this first-ever GWAS of stroke in indigenous Africans, novel genomic regions near genes *AADACL2* and *MIR4458HG* exhibited significant protective associations with ischemic stroke with significant eQTL mapping and chromatin interactions with multiple loci associated with vascular disorders. Our findings identify potential roles of regulatory miRNA, intergenic non-coding DNA, and intronic non-coding RNA in the pathobiology of ischemic stroke among indigenous Africans.

### Supplementary Information

**Additional file 1: Table S1.** Association Results Top Hits <1.0E−06 in Any Model. **Table S2.** Association Results Top Hits<1.0E−04 in Any Model. **Table S3a.** Replication Look-up in COMPASS and MEGASTROKE based on Supplementary Table 1. **Table S3b.** Replication Look-up in COMPASS and MEGASTROKE based on Supplementary Table 2. **Table S4a.** Replication Status of Variants previously associated with Stroke in COMPASS and Comparison with SIREN stroke. **Table S4b.** Replication Status of Variants previously associated with Stroke in MEGASTROKE and Comparison with SIREN stroke. **Table S5a.** Replication of COMPASS significant results in SIREN stroke types. **Table S5b.** Replication of MEGASTROKE significant results in SIREN stroke types. **Table S5c.** COMPASS study’s significant SNPs replication in SIREN for stroke subtypes. **Table S6.** Metasoft meta-analysis results driven by Meta-analysis *P*-value. **Table S7.** Meta-Analysis Results from MANTRA. **Table S8a.** Fine mapping of AADACL2 gene region. **Table S8b.** Posterior Probabilities for SNPs in the AADACL2 fine-mapping regions of gene. **Table S9a.** Fine mapping of MIR4458HG gene region. **Table S9b.** Posterior Probabilities for SNPs in the MIR4458HG fine-mapping regions of gene. **Table S10a.** FUMA GENE2SNP MAGMA differentially expressed genes (DEG) from GTEx v8 data for specific tissue type. **Table S10b.** FUMA GENE2FUNC MAGMA differentially expressed genes (DEG) from GTEx v8 data for specific tissue types. **Table S11.** FUMA GENE2SNP's positional, eQTL or chromatin interaction mapping results on functionally relevant SNPs and GWAS top-hit SNPs. **Table S12.** FUMA SNP2GENE Interactions showing eQTL and Chromatin Interaction statistics based on functionally relevant SNPs and GWAS top-hit SNP with *P*-value < 1E-4 along with Gene and Protein Annotation for selected Tissues. **Table S13a.** Genetic loci associated with stroke by ancestry. **Table S13b.** Functional annotations of genetic loci associated with stroke in MEGASTROKE. **Table S13c.** Functional annotations of genetic loci associated with stroke in COMPASS. **Table S13d.** Functional annotations of genetic loci associated with stroke in GIGASTROKE.

**Additional file 2.** Other Study Cohorts.

**Additional file 3.** Additional Discussion.

**Additional file 4: Fig. S1.** PC1 vs. PC2 plot of genotypes from SIREN and 1000G populations. **Fig. S2.** Manhattan Plots. **Fig. S3.** Locus zoom plots for SNPs rs112549349 (Chr. 2), rs147996143 (Chr. 6), rs2194650 (Chr. 7), rs76534667 (Chr. 12), and rs7326843 (Chr. 13). **Fig. S4.** Scatter plot showing the direction of effect (beta values) between associations of the SNPs with ischemic stroke in both SIREN and COMPASS studies. **Fig. S5.** Comparison of Minor Allele Frequencies among SIREN, COMPASS, and MEGASTROKE. **Fig. S6a.** Tissue gene expression for MIR4458HG using GTExPortal V8. **Fig. S6b.** Tissue gene expression for AADACL2 using GTExPortal V8. **Fig. S7.** (a) FUMA GENE2FUNC differentially expressed genes (DEG) output for 30 general tissue types. (b) FUMA GENE2FUNC differentially expressed genes (DEG) output for 54 specific tissue types. Red bars denote significantly enriched DEG sets (Bonferroni adjusted *P*-values).

## Data Availability

Requests for resources and information should be directed to and will be fulfilled by the lead contact, M. O (mayowaowolabi@yahoo.com). Phenotype and genotype data are available under managed access to researchers. Requests for access will be granted for all research consistent with the consent provided by participants. This would include any research in the context of health and disease that does not involve identifying the participants in any way. The array data have been deposited at the H3Africa Bionet for the European Genome-phenome Archive (accession number: the SIREN study page is: https://ega-archive.org/studies/EGAS00001007331 which contains 2 datasets; phenotype data: EGAD00001011075 https://ega-archive.org/datasets/EGAD00001011075; genotype data: EGAD00010002551 https://ega-archive.org/datasets/EGAD00010002551). The detailed procedure for data access is described according to the H3Africa data access policy available at https://h3africa.org/wp-content/uploads/2020/06/H3Africa-Consortium-Data-Access-Release-Policy-April-2020.pdf. Requests for access to data may be directed to mayowaowolabi@yahoo.com. Applications are reviewed by a data access committee, and access is granted if the request is consistent with the consent provided by participants. The data producers may be consulted by the data access committee to evaluate potential ethical conflicts. Requestors also sign an agreement that governs the terms on which access to data is granted. In addition, the summary GWAS statistics will also be submitted to dbGaP as soon as possible, and the accession number will be provided once available.

## Abbreviations

*AADACL2*: Arylacetamide deacetylase like 2
ARVC5: Arrhythmogenic right ventricular cardiomyopathy
BE: Binary effects model
BFs: Log-Bayes factors
CI: Confidence interval
COMPASS: Consortium of Minority Population GWAS of Stroke
DNA: Deoxyribonucleic acid
eQTLs: Expression quantitative trait loci
FDR: False discovery rate
FE: Fixed effects model
FUMA: Functional Mapping and Annotation
GWAS: Genome-wide association study
H3K4me3: Histone H3 lysine 4 trimethylation
HDL: High-density lipoprotein
*IGSF10*: Immunoglobulin superfamily, member 10
LDL: Low-density lipoprotein
LRT: Likelihood ratio test
LRT: Likelihood ratio test
MAF: Minor allele frequency
MANTRA: Meta-Analysis of TRansethnic Association studies
miRNA: Microribonucleic acid
MRI: Magnetic resonance image
OR: Odd ratio
PC: Principal component
PRS: Polygenic risk scores
QC: Quality control
QQ: Quantile-quantile
QVSFS: Questionnaire for Verifying Stroke-Free Status
RE: Random effects model
RE2: Han and Eskin’s random effects model
SIREN: Stroke Investigative Research and Educational Network
SNP: Single-nucleotide polymorphism
TFBS: Transcription factor binding sites
TMEM43: Transmembrane protein 43
TOAST: Trial of Org 10172 in Acute Stroke Treatment
TOPMEd: Trans-Omics for Precision Medicine
TSS: Transcription start site
UCSC: University of California, Santa Cruz
