# Supplementary material for: Novel functional insights into ischemic stroke biology provided by the first genome-wide association study of stroke in indigenous Africans
Source: Genome Med. 2024 Feb 5;16:25. doi: 10.1186/s13073-023-01273-5 (PMC10840175; doi:10.1186/s13073-023-01273-5)
Supplement: Supplementary file 2 — Additional file 2. Other Study Cohorts. [file 13073_2023_1273_MOESM2_ESM.docx]

**OTHER STUDY COHORTS**

**The MEGASTROKE Consortium**

The MEGASTROKE Consortium (521,612 individuals: 67,162 cases and 454,450 controls) consists of several studies including the METASTROKE consortiumly^1^, NINDS-SIGN consortium^2^ and the CHARGE consortium.

The constituent studies of the CHARGE Consortium[^3^](#_ENREF_13) include:

***Age, Gene/Environment Susceptibility (AGES) -Reykjavik Study***

The AGES-Reykjavik Study is a single center prospective cohort study based on the Reykjavik Study. The Reykjavik Study was initiated in 1967 by the Icelandic Heart Association to study cardiovascular disease and risk factors.[^4^](#_ENREF_14) The cohort included men and women born between 1907 and 1935 who lived in Reykjavik at the 1967 baseline examination. Re-examination of surviving members of the cohort was initiated in 2002 as part of the AGES-Reykjavik Study. The AGES-Reykjavik Study is designed to investigate aging using a multifaceted comprehensive approach that includes detailed measures of brain function and structure. All cohort members were European Caucasians. Briefly, as part of a comprehensive examination, all participants answered a questionnaire, underwent a clinical examination and had blood drawn. Among AGES participants with GWAS data (N=3,219), after exclusion of participants with prevalent stroke (N=224), and those without follow-up for incident stroke events (N=114), N=2,996 participants were available for analyses.

*Stroke ascertainment in AGES*: Incident stroke cases were ascertained from multiple sources including hospital, general practice, nursing home records and death certificates. All possible cases were adjudicated with standard TOAST criteria by two Neurologists and a Neuroradiologist with expertise in evaluating stroke cases for epidemiologic studies.

***Atherosclerosis Risk in Communities (ARIC) Study***

The ARIC study is a prospective population-based study of atherosclerosis and clinical atherosclerotic diseases in 15,792 men and women, including 11,478 non-Hispanic white participants, drawn from 4 U.S. communities (Suburban Minneapolis, Minnesota; Washington County, Maryland; Forsyth County, North Carolina, and Jackson, Mississippi). In the first three communities, the sample reflects the demographic composition of the community. In Jackson, only black residents were enrolled. Ancestry was self-reported during an interview. Participants were handed a card and asked to tell the interviewer which best described his or her race. Choices offered were: White, Black, American Indian/Alaskan Native, Asian/Pacific Islander, Other: specify. Over 99% identified as either white or black. Only self-identified blacks were included in for COMPASS. Participants were between age 45 and 64 years at their baseline examination in 1987-1989 when blood was drawn for DNA extraction and participants consented to genetic testing. Only individuals free of stroke or TIA at baseline were included in the analysis. Single-nucleotide polymorphisms (SNPs) were genotyped on the Affymetrix 6.0 chip and were imputed to ≈2.5 million SNPs based on a panel of cosmopolitan reference haplotypes from HapMap CEU and YRI (HapMap II CEU and YRI (build 35, release 21)). MACH v1.0.16 was used to perform genotype imputations and allele dosage information was summarized in the imputation results.

*Stroke ascertainment in ARIC:* Hospitalized strokes that occurred by December 31, 2016 were included in the present study. During annual telephone contacts, trained interviewers asked each ARIC participant to list all hospitalizations during the past year. Hospital records for any hospitalizations identified were then obtained. In addition, all local hospitals annually provided lists of stroke discharges (International Classification of Diseases, Ninth Revision, Clinical Modification codes 430 to 438), which were scrutinized for ARIC participant discharges. Details on quality assurance for ascertainment and classification of stroke are described elsewhere. Briefly, the stroke diagnosis was assigned according to criteria adapted from the National Survey of Stroke. Strokes secondary to trauma, neoplasm, hematologic abnormality, infection, or vasculitis were excluded, and a focal deficit lasting <24 hours was not considered to be a stroke. Out-of-hospital stroke was not ascertained and validated; thus, these potential stroke events were not included. Strokes were classified into hemorrhagic stroke (subarachnoid and intracerebral hemorrhage) and ischemic stroke (thrombotic and embolic brain infarction). A stroke was classified as ischemic when a brain CT or MRI revealed acute infarction and showed no evidence of hemorrhage. All definite ischemic strokes were further classified as lacunar, nonlacunar thrombotic, or cardioembolic on the basis of the recorded neuroimaging results. For this analysis, the hemorrhagic strokes identified by ARIC were censored at the time of their occurrence.

***Cardiovascular Health Study (CHS) – European Ancestry***

The Cardiovascular Health Study (CHS) is a population-based cohort study of risk factors for coronary heart disease and stroke in adults ≥65 years conducted across four field centers.[^5^](#_ENREF_15) The original predominantly European ancestry cohort of 5,201 persons was recruited in 1989-1990 from random samples of people on the Medicare eligibility lists; subsequently, an additional predominantly African-American cohort of 687 persons was enrolled for a total sample of 5,888. Blood samples were drawn from all participants at their baseline examination and DNA was subsequently extracted from available samples. Because the other cohorts in the CHARGE analysis were predominantly European ancestry, the African American participants were excluded from this analysis to reduce the possibility of confounding by population structure. European ancestry participants were excluded from this GWAS study sample due to the presence at study baseline of coronary heart disease, congestive heart failure, peripheral vascular disease, valvular heart disease, stroke or transient ischemic attack, or lack of available DNA. Beyond laboratory genotyping failures, participants were excluded if they had a call rate<=95% or if their genotype was discordant with known sex or prior genotyping. After quality control, genotyping was successful for 3,268 European ancestry participants. CHS was approved by institutional review committees at each field center and individuals in the present analysis had available DNA and gave informed consent including consent to use of genetic information for the study of cardiovascular disease.

*Stroke ascertainment in CHS*: Participants were examined annually from enrollment to 1999 and continued to be under surveillance for stroke following 1999.[^6^](#_ENREF_16)^,^[^7^](#_ENREF_17) Since baseline, participants have also been contacted twice a year to identify potential cardiovascular events, including stroke. In addition, all hospitalizations were screened for potential stroke events. For suspected fatal and non-fatal events occurring with or without hospitalization, information was collected from the participant or next of kin, from medical records, and, if needed, from the participant's physician. When available, scans or reports of CT, MRI or both were reviewed centrally. Final at a consensus conference using all available information vascular neurologists adjudicated the occurrence of fatal and non-fatal stroke, stroke types, and subtypes. Stroke definitions were derived from the criteria used for the Systolic Hypertension in the Elderly Program (SHEP).[^8^](#_ENREF_18) Stroke types were ischemic, hemorrhagic and other based on brain imaging. Hemorrhagic stroke subtypes were intra-parenchymal, subarachnoid, and other. Ischemic stroke subtypes were 1) small vessel, 2) large vessel, 3) cardioembolic, and 4) other that included mostly uncertain subtypes. The approach used in CHS was developed before the TOAST criteria were published in 1993.[^9^](#_ENREF_19) Nonetheless, the two approaches are quite similar.

***Framingham Heart Study (FHS)***

The Framingham Heart Study (FHS) is a three-generation, single-site, community-based, ongoing cohort study that was initiated in 1948 to investigate prospectively the risk factors for CVD including stroke. It now comprises 3 generations of participants (N=10,333): the Original cohort followed since 1948;[^10^](#_ENREF_20) their Offspring and spouses of the Offspring, followed since 1971;[^11^](#_ENREF_21) and children from the largest Offspring families enrolled in 2000 (Gen 3).[^12^](#_ENREF_22) Gen 3 participants were not included in this analysis since they are young (mean age 40±9 years) and few have suffered strokes. The Original cohort enrolled 5209 men and women who comprised two-thirds of the adult population then residing in Framingham, MA. Survivors continue to receive biennial examinations. The Offspring cohort comprises 5124 persons (including 3514 biological offspring) who have been examined approximately once every 4 years. The population of Framingham was virtually entirely white (Europeans of English, Scots, Irish and Italian descent) in 1948 when the Original cohort was recruited. At the initial examination participants were asked for country of birth and whether or not they had any Italian ancestry. At a later examination (the 8^th^) the Offspring cohort participants were asked to identify their race from the following choices: Caucasian or white, African-American or black, Asian, Native Hawaiian or other Pacific Islander, American Indian or Alaska native or ‘prefer not to answer’. They were either asked to identify their ethnicity as either ‘Hispanic or Latino’ or not. Almost all the FHS Original and Offspring participants are white/Caucasian and none were excluded from the discovery cohort. FHS participants had DNA extracted and provided consent for genotyping in the 1990s. All available eligible participants underwent genome-wide genotyping. In 272 persons (31 with stroke), small amounts of DNA were extracted from stored whole blood and required whole genome amplification prior to genotyping. Cell lines were available for most of the remaining participants.

Among FHS participants with GWAS data (N=4,535), after exclusion of participants with prevalent stroke (N=138), and those without follow-up for incident stroke events (N=12), N=4,385 participants were available for analyses.

*Stroke ascertainment in FHS:* At each clinic exam, participants receive questionnaires, physical examinations and laboratory testing; between examinations they remain under surveillance (regardless of whether or not they live in the vicinity) via physician referrals, record linkage and annual telephone health history updates. Incident strokes have been identified since 1948 through this ongoing system of FHS clinic and local hospital surveillance and methods used have been detailed previously;[^13-15^](#_ENREF_23) they include review of medical records and collaboration with local general practitioners, emergency rooms and imaging facilities. If a participant saw a physician or was admitted to the hospital, visited an emergency room or obtained any brain imaging between biennial examinations for symptoms suggestive of TIA or stroke, a stroke neurologist from the Heart Study attempted to visit the person within 48 hours and recorded a complete history and neurological examination; this was repeated at 1, 3 and 6 months. All medical records from practitioners, hospitals, imaging centers, rehabilitation centers and nursing homes were procured for review. A panel of 3 investigators (at least 2 neurologists) adjudicated the diagnosis of stroke and determined stroke subtype in each case based on the Framingham evaluations and external records. The recruitment of Original and Offspring cohort participants at FHS had occurred long before the DNA collection with the result that the majority of stroke events in the FHS (although ascertained prospectively) were prevalent at the time of DNA collection and were excluded from these analyses. While this reduced the sample size from FHS, the meta-analyses presented here focused on incident events.

***FINRISK***

FINRISK surveys are cross-sectional, population-based studies conducted every 5 years since 1972 to monitor the risk of chronic diseases. For each survey, a representative random sample was selected from 25- to 74-year-old inhabitants of different regions in Finland. The survey included a questionnaire and a clinical examination, at which a blood sample was drawn, with linkage to national registers of cardiovascular and other health outcomes. The study protocol has been described elsewhere.[^16^](#_ENREF_26) The current study included eligible individuals from FINRISK surveys conducted in 1992, 1997, 2002, and 2007.

*Stroke ascertainment in FINRISK:* During follow-up, participants were monitored for stroke through linkage of the study database with the National Hospital Discharge Register and the National Causes-of-Death Register. The clinical outcomes were linked to study subjects using their unique national social security ID, which is assigned to every permanent resident of Finland. The registers are countrywide covering all cardiovascular events that have led either to hospitalization or death in Finland. Their stroke diagnoses have been validated.[^17^](#_ENREF_27) With both registers the diagnostic classification was done using the Finnish adaptation of ICD-codes: I63; not I63.6, I64 (ICD-10) / 4330A, 4331A, 4339A, 4340A, 4341A, 4349A, 436 (ICD-9) / 433, 434, 436 (ICD-8) for Ischemic stroke excluding any hemorrhagic strokes, and I60-I61,I63-I64 (not I63.6) (ICD-10) / 430, 431, 4330A, 4331A, 4339A, 4340A, 4341A, 4349A, 436 (ICD-9) / 430, 431 (except 431.01, 431.91), 433, 434, 436 (ICD-8) for all-stroke including SAH. ICD-8 codes 430, 431 (excluding codes 431.01, 431.91 of the Finnish adaptation of ICD-8*), 432, 433, 434 or with ICD-9 codes 430, 431, 433 (excluding codes 4330X, 4331X, 4339X of the Finnish adaptation of ICD-9*), 434 (excluding code 4349X of the Finnish adaptation of ICD-9*), 436, 437, 438 or with ICD-10 codes I60, I61, I63 (excluding I63.6), I64 or I69.[^18^](#_ENREF_28) The stroke was classified as a first-ever event if there was no evidence of a previous stroke event in the patient’s history. An event found in either register was sufficient for diagnosis.

***Health, Aging, and Body Composition (Health ABC) Study***

The Health ABC study is a prospective cohort study designed to examine the associations between body composition, weight-related health conditions, and functional limitations in older adults aged 70-79 years at inception.[^19^](#_ENREF_29) In 1997-1998, 3,075 participants were recruited from a random sample of white and all African-American Medicare eligible residents in the Pittsburgh, PA and Memphis, TN metropolitan areas. Genome-wide genotyping was performed in 1732 white participants and 1663 met all QC criteria. All participants provided informed consent and protocols were approved by the institutional review boards at both study sites.

Among Health ABC participants with GWAS data (N=1663), after exclusion of participants without follow-up for incident stroke events (N=2), N=1,661 participants were available for analyses.

*Stroke ascertainment in Health, Aging, and Body Composition (Health ABC) Study:* Participants were screened for stroke events every 6 months alternating between semi-annual phone interviews and annual clinical visits. Any self-reported hospitalization for stroke led to medical record abstraction and verification by a Health ABC Disease Adjudicator at each site. Date and causes of death were obtained from the death certificate. Causes of death were adjudicated based on the review of medical records, proxy information and autopsy report (when performed).[^20^](#_ENREF_30)^,^[^21^](#_ENREF_31) Stroke subtyping was done from medical records review.  If the medical record indicated the event was hemorrhagic or ischemic in nature, this was recorded in the Health ABC data.

***Rotterdam Study***

The Rotterdam Study is a population-based cohort study among inhabitants of a district of Rotterdam (Ommoord), The Netherlands, and aims to examine the determinants of disease and health in the elderly with a focus on neurogeriatric, cardiovascular, bone, and eye disease.[^22-24^](#_ENREF_32) All inhabitants aged ≥55 years (N=10,275) were invited and the participation rate was 78%, yielding a total of 7983 subjects. All participants gave written informed consent to retrieve information from treating physicians. Baseline measurements were obtained from 1990 to 1993 and consisted of an interview at home and two visits to the research center for physical examination. At this baseline examination ancestry was determined by self-report. Participants were asked to identify with one of the following categories that best described their ancestry: Dutch, Caucasian, Asian, Indian, Indonesian, Mediterranean, Negroid. Less than 1% of participants chose an ancestry other than Dutch or Caucasian. Survivors have been re-examined three times: in 1993-1995, 1997-1999, and 2002-2004. All persons attending the baseline examination in 1990-93 consented to genotyping and had DNA extracted. Genome-wide genotyping was attempted in persons with high-quality extracted DNA.

In 1990-1993, 7 983 persons 55 years of age or over participated and were re-examined every 3 to 4 years. In 1999, 3 011 individuals who had become 55 years of age or moved into the study district since the start of the study were added to the cohort (Rotterdam Study-II).[^23^](#_ENREF_33) All participants had DNA extracted at their first visit. Genotyping was attempted in participants with high-quality extracted DNA.

Among Rotterdam Study-I participants with GWAS data (N=6,291), after exclusion of participants with prevalent stroke (N=179), and those without follow-up for incident stroke events (N=46), N=6,066 participants were available for analyses.

Among Rotterdam Study-II participants with GWAS data (N=2,157), after exclusion of participants with prevalent stroke (N=76), and those without follow-up for incident stroke events (N=1), N=2,080 participants were available for analyses.

*Stroke ascertainment in Rotterdam:* All participants have been continuously monitored for major events (including stroke) through automated linkage of the study database with files from general practitioners and the municipality. In addition physician files from nursing homes and general practitioner records of participants who moved out of the Ommoord district were reviewed twice a year. For suspected stroke and TIA events, both fatal and non-fatal, additional information (including neuroimaging) was obtained from general practitioner’ and hospital records and research physicians discussed available information with an experienced stroke neurologist to verify all diagnoses and to subclassify the strokes. Strokes were subclassified into ischemic or hemorrhagic based on neuroimaging (CT or MRI within 3 weeks) mentioned in medical records. If a hemorrhage was shown the stroke was subclassified as hemorrhagic, if there were no signs of hemorrhage, the stroke was subclassified as ischemic. Furthermore, strokes were subclassified according to TOAST criteria based on the diagnostic workup mentioned in medical records.[^25^](#_ENREF_35)^,^[^26^](#_ENREF_36)

***Study of Health in Pomerania (SHIP)***

The “Study of Health in Pomerania” is a population-based epidemiological study in the region of Western Pomerania, Germany.[^27^](#_ENREF_37) In brief, from the total population of West Pomerania comprising 213 057 inhabitants in 1996, a two-stage stratified cluster sample of adults aged 20–79 years was drawn. The net sample (without migrated or deceased persons) comprised 6 265 eligible subjects, out of which 4 308 completed their baseline examinations. From July 2007 to October 2010 the ‘Life-Events and Gene-Environment Interaction in Depression’ (LEGENDE) study was carried out in the SHIP cohort. After exclusion of SHIP- 1 participants without GWAS data and a positive lifetime prevalence of stroke before the SHIP-1 examination (N=188), N=3,112 participants were available for analyses.

*Stroke ascertainment in SHIP:* SHIP participants were followed-up after a median (range) of 5.0 (4.3–8.5) years on average. New stroke events were identified based on the following sources: Self-report by participants during the follow-up visit at the clinic center, with specific questions asking for a physician diagnosis (self-reported physician's diagnosis of stroke);[^28^](#_ENREF_38) ICD codes based on the statutory health insurance, a survey among family doctors, inpatient visits at the Greifswald University Hospital, and Death Certificates. We included cases with fatal and non-fatal strokes. For in- and outpatient data we defined any stroke as cases with a coded ICD I61, I63, I64, I69.1, I69.3, I69.4 diagnosis. For ischemic stroke we included all cases with I63.x codes based on in- and outpatient data. Data from participants with self-reported events lacking an external validation were right censored at the estimated date of event. All participants with any stroke event from any source before the baseline examination were excluded from analyses.

***Women’s Genome Health Study (WGHS)***

The WGHS (Women’s Genome Health Study) is a large cohort for genome-wide genetic analysis of a wide range of clinical phenotypes among >25 000 women, 45 years or older at baseline and with ongoing follow-up observation, now for approximately 18 years.[^29^](#_ENREF_39) The population is derived from participants in the Women’s Health Study (WHS) who provided a blood sample at baseline. By design, participants included in the WGHS were free from dementia and stroke at baseline. Similarly, follow-up for incident stroke events was complete in the WGHS. Therefore, the total number of WGHS participants with whole genome genetic data for analysis was N=23,294.

*Stroke ascertainment in WGHS:*Since enrollment WGHS participants were followed-up annually for the occurrence of relevant clinical endpoints including stroke. The end-point ascertainment was continued in a blinded fashion through the scheduled end of the trial (March 31, 2004), when the cohort was converted to observational mode. Follow-up and validation of reported end points continues through the ongoing observational period. When a stroke endpoint was reported to occur, full medical reports were obtained and reviewed by an endpoints committee of physicians unaware of randomized treatment assignment. A confirmed stroke was defined as a new neurologic deficit of sudden onset that persisted for >24 h. Clinical information as well as computed tomographic scans or MRI were used to distinguish hemorrhagic from ischemic events.[^29^](#_ENREF_39) Stroke subtyping definition distinguishes ischemic versus hemorrhagic events according to TOAST criteria.[^9^](#_ENREF_19)

***Multi-Ethnic Study of Atherosclerosis (MESA)***

The Multi-Ethnic Study of Atherosclerosis (MESA) is a study of the characteristics of subclinical cardiovascular disease (disease detected non-invasively before it has produced clinical signs and symptoms) and the risk factors that predict progression to clinically overt cardiovascular disease or progression of the subclinical disease.[^30^](#_ENREF_40) MESA researchers study a diverse, population-based sample of 6,814 asymptomatic men and women aged 45-84. Thirty-eight percent of the recruited participants are white, 28 percent African-American, 22 percent Hispanic, and 12 percent Asian, predominantly of Chinese descent. Only white participants were used for the present analysis.

Participants were recruited from six field centers across the United States: Wake Forest University, Columbia University, Johns Hopkins University, University of Minnesota, Northwestern University and University of California - Los Angeles. The first examination took place over two years, from July 2000 - July 2002. It was followed by four examination periods that were 17-20 months in length. Participants have been contacted every 9 to 12 months throughout the study to assess clinical morbidity and mortality.[^30^](#_ENREF_40)

Prevalent stroke was an exclusion criterion for MESA at baseline. Among MESA white participants with GWAS data (N=2,685), we excluded those with unexpected ancestry as inferred by principal components (N=124), those with unexpected relatedness (N=35), and those without follow-up for incident stroke events (N=162), N=2,364 participants were available for analyses.

*Stroke ascertainment in MESA:* New occurrences of stroke were recorded over 7-years of follow-up. In brief, a telephone interviewer contacted each participant every 9–12 months. Information about all new cardiovascular conditions, hospital admissions, cardiovascular outpatient diagnoses, treatments, and deaths were obtained. To verify self-reported diagnoses, information was collected from death certificates and medical records for all hospitalizations and outpatient cardiovascular diagnoses, using ICD-9 and ICD-10 codes. In the case of out-of-hospital deaths, next-of-kin interviews or questionnaires were administered to physicians, relatives or friends. Two physicians from the MESA study events committee independently reviewed all medical records for end point classification and assignment of incidence dates. The reviewers were blinded to the study data. If the reviewing physicians disagreed on the event classification, they adjudicated differences. Neurologists reviewed and classified stroke as present if there was a focal neurologic deficit lasting 24 hours or until death, or if <24h, there was a clinically relevant lesion on brain imaging and no nonvascular cause. Patients with focal neurological deficits secondary to brain trauma, tumor, infections, or other non-vascular cause were excluded.[^31^](#_ENREF_41) Ischemic strokes were distinguished from hemorrhagic stroke using findings on imaging, surgery, autopsy, or some combination of these. Ischemic stroke subtypes were assigned based on an extension of the Trial of Org 10172 in Acute Stroke Treatment (TOAST) scheme to try to reduce the number classified as undetermined.

***PROspective Study of Pravastatin in the Elderly at Risk (PROSPER)***

PROSPER was a prospective multicenter randomized placebo-controlled trial to assess whether treatment with pravastatin diminishes the risk of major vascular events in elderly. Between December 1997 and May 1999, we screened and enrolled subjects in Scotland (Glasgow), Ireland (Cork), and the Netherlands (Leiden). Men and women aged 70-82 years were recruited if they had pre-existing vascular disease or increased risk of such disease because of smoking, hypertension, or diabetes. A total number of 5804 subjects were randomly assigned to pravastatin or placebo. A large number of prospective tests were performed including Biobank tests and cognitive function measurements. A detailed description of the study has been published elsewhere. ^32,33^

Among PROSPER participants with GWAS data (N=5,244), after exclusion of participants with prevalent stroke (N=586), and those without follow-up for incident stroke events (N=0), N=4,658 participants were available for analyses.

*Stroke ascertainment in PROSPER:* Stroke was defined as any event that meets the criteria listed below:

(a) Ischemic stroke (1 of the following conditions must be met): (1) Rapid onset of focal neurologic deficit lasting >24 hours or leading to death plus evidence from neuroimaging (computed tomography or magnetic resonance imaging) showing cerebral/cerebellar infarction or no abnormality, or postmortem examination showing cerebral and/or cerebellar infarction. (2) Rapid onset of global neurologic deficit (e.g., coma) lasting >24 hours or leading to death plus evidence from neuroimaging showing infarction, or postmortem examination showing infarction. (3) Focal neurologic deficit (mode of onset uncertain) lasting >24 hours or leading to death plus evidence from neuroimaging showing infarction, or postmortem examination showing infarction.

(b) Primary intracerebral and/or cerebellar hemorrhage (1 of the following conditions must be met): (1) Rapid onset of focal neurologic deficit lasting >24 hours or leading to death, plus neuroimaging or postmortem examination showing primary intracerebral and/or cerebellar hemorrhage. (2) Rapid onset of global neurologic deficit (e.g., coma) lasting >24 hours or leading to death, plus evidence from neuroimaging or postmortem examination showing primary intracerebral and cerebellar hemorrhage. (3) Focal neurologic deficit (mode of onset uncertain) lasting >24 hours or leading to death, plus evidence from neuroimaging or postmortem examination showing primary intracerebral and/or cerebellar hemorrhage.

(c) Not known (1 of the following conditions must be met): (1) Rapid onset of focal neurologic deficit lasting >24 hours or leading to death, without neuroimaging or postmortem data available. (2) Rapid onset of global neurologic deficit (e.g., coma) lasting >24 hours or leading to death, without neuroimaging or postmortem data available. (3) Focal neurologic deficit (mode of onset uncertain) lasting >24 hours or leading to death, without neuroimaging or postmortem data available.

The PROSPER Endpoints Committee was responsible for the classification of all possible study end points. The Committee received all annual study electrocardiograms showing serial changes, information regarding domiciliary visits or hospitalizations associated with possible myocardial infarction, and information on all deaths (including postmortem reports, death certificates, hospital records, general practitioners’ records, and/or interviews of family members or witnesses).

***3C-Study***

The Three-City study is a prospective study aiming to assess the association between vascular diseases and risk of dementia. The detailed protocol of the study has been previously described.[^34^](#_ENREF_44) The Three-City cohort is composed of non-institutionalized individuals aged 65 years and over, randomly selected from electoral rolls of three cities of France (Bordeaux, Dijon, and Montpellier), and agreeing to participate in the study. Between March 1999 and March 2001, 9,294 persons were enrolled (4,931 in Dijon, 2,104 in Bordeaux and 2,259 in Montpellier).

Up to five face-to-face examinations were performed during follow-up. Trained nurses and psychologists performed interviews and physical and cognitive measurements at the participant’s home and at the study centre. As imputation was performed separately in the 3C-Dijon sample on the one hand and the Bordeaux and Montpellier samples on the other hand, analyses were run separately in these datasets (3C-Dijon and 3C-Bordeaux-Montpellier).

In the 3C-Dijon study, among participants with GWAS data (N=4,077), after exclusion of participants with prevalent stroke (N=204), and those without follow-up for incident stroke events (N=111), N=3,762 participants were available for analyses.

In the 3C-Bordeaux-Montpellier study, after exclusion of participants without GWAS data and with prevalent stroke and those without follow-up for incident stroke events N=2,153 participants were available for analyses.

*Stroke ascertainment in 3C-Study:* At each follow-up visit, participants or informants for deceased participants were systematically questioned about the occurrence of any severe medical event or hospitalization since the last contact. For those reporting a possible stroke event, all available clinical information was collected from hospital records, and interviews with the participant’s physician, nursing home staff (for participants admitted in a nursing home during follow-up) or family. Expert panels including at least one physician specialized in vascular medicine reviewed all available clinical information and classified each event according to the International Classification of Diseases – 10^th^ Edition. Stroke was confirmed if the participant had a new focal neurological deficit of sudden onset attributable to a cerebrovascular event that persisted for more than 24 hours. Stroke was classified by the panel as ischemic stroke, intracerebral hemorrhage or of unspecified type and ischemic stroke (IS) was classified by the panel according to the TOAST classification into cardioembolic IS, large-artery IS, small vessel disease IS, IS of other etiologies, and IS of undetermined etiology.[^9^](#_ENREF_19)

**EPIC-CVD**

EPIC is a multi-centre prospective cohort study of 519,978 participants (366,521 women and 153,457 men, mostly aged 35–70 years) recruited between 1992 and 2000 in 23 centres located in 10 European countries.[^35^](#_ENREF_45) Participants were invited mainly from population-based registers (Denmark, Germany, certain Italian centres, the Netherlands, Norway, Sweden, UK). Other sampling frameworks included: blood donors (Spain and Turin and Ragusa in Italy); screening clinic attendees (Florence in Italy and Utrecht in the Netherlands); people in health insurance programmes (France); and health conscious individuals (Oxford, UK). About 97% of the participants were of white European ancestry. Prevalent CVD was ascertained through self-reported history of MI or angina, or registry-ascertained CVD event prior to baseline. EPIC-CVD employs a nested case-cohort design,[^36^](#_ENREF_46) analogous to the EPIC-InterAct study for type-2 diabetes which established a common set of referents through selection of a random sample of the entire cohort (“subcohort”).[^37^](#_ENREF_47)

*Stroke ascertainment in EPIC:* Centres were asked to ascertain suspected stroke cases from registries, hospital records or self-report (i.e. follow-up questionnaires). Stroke events were defined by ICD10 codes as follows: Ischemic I63, Haemorrhagic I61, SAH I60, Unclassified I64, Other CRBV I62, I65-I69, F01. Incident stroke cases have been defined as fatal and non-fatal. All centres have recorded cause-specific mortality through mortality registries and/or active follow-up, and have ascertained and validated incident fatal and non-fatal stroke through a combination of methods.

Ascertained non-fatal stroke events were validated by clinical symptoms and imaging evidence (CT/MRI) or confirmed through hospital/GP records (with assessment of notes) or confirmed through hospital records (without assessment of notes). Individuals were excluded if they had clinical symptoms but no validation was possible e.g. there was no imaging evidence, nor GP/primary care records (without assessment of notes) or registry information. Fatal stroke events were validated either by autopsy or hospital records and death certificate or by death certificate if they died in hospital. Individuals where validation was not possible were excluded. Participants with a history of stroke or MI at baseline were excluded. No further stroke subtyping was performed.

**Biobank Japan**

BioBank Japan Project (BBJ) was started in 2003 and collected DNA and clinical information from about 200,000 patients with 47 common diseases between 2003 and 2007 (BBJ 1st cohort), and about 67,000 patients with 38 diseases between 2013 and 2017 (BBJ 2nd cohort).

Eligibility of cases was determined by physicians from a collaborative network of 66 hospitals. Individuals were excluded from the analysis if they were not supposed to be East Asian ancestry, genetic sex discordance, or with other possible clinical information errors. Overlapping individuals between BBJ cohorts and in-house imputation panel were excluded before imputation. Among the 177,261 subjects genotyped from BBJ 1st cohort after QC, 17,651 cases were registered for ischemic stroke. Of all ischemic stroke cases, 1,331 were classified as large artery strokes, 758 as cardioembolic and 4,915 as small vessel stroke. Clinical information was collected by standardized questionnaire though medical records survey. Independent genotype data of 9,809 subejects from BBJ 1st cohort, and 41,929 subjects from BBJ 2nd cohort were used for the validation of polygenic risk score. 577 and 1,470 ischemic stroke cases were registered, respectively.

*Stroke ascertainment in Biobank Japan:* Stroke patients were selected from BioBank Japan. Ischemic stroke was diagnosed by physicians at collaborating hospitals and its subtypes were determined by medical record survey according to the TOAST criteria. For GWAS, individuals without any stroke or intracranial aneurysm were selected as controls ( n=159,610).

**CADISP**

The Cervical Artery Dissections and Ischemic Stroke Patients (CADISP) study was designed to identify genetic risk variants for cervical artery dissections (CeAD), a major cause of ischemic stroke in young adults.[^48^](#_ENREF_48) As part of a secondary analysis, patients with an ischemic stroke without cervical artery dissection (non-CeAD ischemic stroke) were also recruited, in the same centers as CeAD patients. These were patients with a diagnosis of ischemic stroke, in whom CeAD had been formally ruled out according to CADISP inclusion criteria (see attachment). Non-CeAD ischemic stroke patients were frequency-matched on age (by 5-year intervals) and gender on CeAD patients. A total of 658 non-CeAD ischemic stroke patients were included in Belgium, Finland, France, Germany, Italy, and Switzerland. We excluded 19 patients due to unavailability of geographically matched healthy controls, or due to non-European origin; of the remaining 639 non-CeAD IS patients, 613 individuals had good quality DNA available and were genotyped at the CNG. Of these, a total of 555 non-CeAD IS patients aged < 60 years, who were successfully genotyped and met genotyping quality control criteria, were used for the present analysis.

The abstracted hospital records of cases were reviewed and adjudicated for IS subtype by a neurologist in each participating center. Each item required for the subtype classification was also recorded in a standardized fashion. Based on this, IS subtypes were then centrally re-adjudicated by a panel of neurologists, in agreement with the TOAST system,[^9^](#_ENREF_19) using a more detailed subtype description from an early version of the Causative Classification System (CCS).[^39^](#_ENREF_49)

The majority of controls (N=9,046, of which 74 Finns and 8,972 non-Finnish Europeans) were selected from an anonymized control genotype database at the Centre National de Génotypage [CNG], in order to match cases for ethnic background, based on principal component analysis. European reference samples from the genotype repository at the CNG were also analyzed simultaneously to provide improved geographical resolution. Additional Finnish controls were recruited within the CADISP study, both from the general population and among spouses and unrelated friends of CADISP patients, within the Helsinki area. A total of 234 individuals were eligible for genotyping at the CNG. Of these, 213 individuals who were genotyped successfully and met quality control criteria were available for the present analysis.[^38^](#_ENREF_48) All participants were of European ancestry.

**THE COMPASS CONSORTIUM**

(physician-adjudicated stroke patients =3734 and no history of stroke = 18317)

COMPASS contains African American participants from several cohort studies. Diagnosis of stroke was adjudicated by a physician. The study has been described previously,40 details on the individual cohorts are as follows.

Atherosclerosis Risk in Communities (ARIC) Study

The ARIC study is a prospective population-based study of atherosclerosis and clinical atherosclerotic diseases in 15,792 en and women, including 11,478 non-Hispanic white participants, drawn from 4 U.S. communities (Suburban Minneapolis, Minnesota; Washington County, Maryland; Forsyth County, North Carolina, and Jackson, Mississippi). In the first three communities, the sample reflects the demographic composition of the community. In Jackson, only black residents were enrolled. Ancestry was self-reported during an interview. Participants were handed a card and asked to tell the interviewer which best described his or her race. Choices offered were: White, Black, American Indian/Alaskan Native, Asian/Pacific Islander, Other: specify. Over 99% identified as either white or black. Only self-identified blacks were included in for COMPASS. Participants were between age 45 and 64 years at their baseline examination in 1987-1989 when blood was drawn for DNA extraction and participants consented to genetic testing. Only individuals free of stroke or TIA at baseline were included in the analysis. Single-nucleotide polymorphisms (SNPs) were genotyped on the Affymetrix 6.0 chip and were imputed to ≈2.5 million SNPs based on a panel of cosmopolitan reference haplotypes from HapMap CEU and YRI (HapMap II CEU and YRI (build 35, release 21)). MACH v1.0.16 was used to perform genotype imputations and allele dosage information was summarized in the imputation results.

Stroke ascertainment in ARIC: Hospitalized strokes that occurred by December 31, 2012 were included in the present study. During annual telephone contacts, trained interviewers asked each ARIC participant to list all hospitalizations during the past year. Hospital records for any hospitalizations identified were then obtained. In addition, all local hospitals annually provided lists of stroke discharges (International Classification of Diseases, Ninth Revision, Clinical Modification codes 430 to 438), which were scrutinized for ARIC participant discharges. Details on quality assurance for ascertainment and classification of stroke are described elsewhere. Briefly, the stroke diagnosis was assigned according to criteria adapted from the National Survey of Stroke. Strokes secondary to trauma, neoplasm, hematologic abnormality, infection, or vasculitis were excluded, and a focal deficit lasting <24 hours was not considered to be a stroke. Out-of-hospital stroke was not ascertained and validated; thus, these potential stroke events were not included. Strokes were classified into hemorrhagic stroke (subarachnoid and intracerebral hemorrhage) and ischemic stroke (thrombotic and embolic brain infarction). A stroke was classified as ischemic when a brain CT or MRI revealed acute infarction and showed no evidence of hemorrhage. All definite ischemic strokes were further classified as lacunar, nonlacunar thrombotic, or cardioembolic on the basis of the recorded neuroimaging results. For this analysis, the hemorrhagic strokes identified by ARIC were censored at the time of their occurrence.

Cardiovascular Health Study (CHS) – African-Americans

The Cardiovascular Health Study (CHS) is a population-based cohort study of risk factors for coronary heart disease and stroke in adults ≥65 years conducted across four field centers .15 The original predominantly European ancestry cohort of 5,201 persons was recruited in 1989-1990 from random samples of people on the Medicare eligibility lists; subsequently, an additional predominantly African-American cohort of 687 persons were enrolled for a total sample of 5,888. Because the COMPASS consortium focused on non-European samples, only self-described African-Americans contributed to the COMPASS analyses.

Blood samples were drawn from all participants at their baseline examination and DNA was subsequently extracted from available samples. Genotyping was performed at the General Clinical Research Center’s Phenotyping/Genotyping Laboratory at Cedars-Sinai among CHS African-American participants who consented to genetic testing and had DNA available using Illumina HumanOmni1-Quad_v1 BeadChip system.

Beyond laboratory genotyping failures, participants were excluded if they had a call rate<=95% or if their genotype was discordant with known sex or prior genotyping (to identify possible sample swaps). After quality control, genotyping was successful for 823 African-American participants.

CHS was approved by institutional review committees at each field center and individuals in the present analysis had available DNA and gave informed consent including consent to use of genetic information for the study of cardiovascular disease.

The Healthy Aging in Neighborhoods of Diversity across the Life Span Study (HANDLS) – African Americans

In the absence of non-stroke control samples from the VISP, ISGS, and SWISS studies, controls from the Healthy Aging in Neighborhoods of Diversity across the Life Span study (HANDLS) study were used for the VISP and SWISS-ISGS case-control analyses (with no overlap across studies). Controls were sex and race/ethnicity-matched and randomly selected from all HANDLS participants not reporting history of stroke at baseline or reporting adjudicated stroke during follow-up.

HANDLS is an interdisciplinary, community-based, prospective longitudinal epidemiologic study examining the influences of race and socioeconomic status (SES) on the development of age-related health disparities among socioeconomically diverse African Americans and whites in Baltimore, MD, USA. This study assesses physical parameters over a 20-year period while evaluating genetic, biologic, demographic, and psychosocial influences. HANDLS recruited 3,722 participants (2200 African Americans (59%) and 1522 whites (41%)) from Baltimore, MD.

Stroke Ascertainment. Stroke status at baseline was determined through self-report while incident strokes, other vascular events, and deaths were determined using medical records and clinic visits during follow-up.

Genotyping was focused on a subset of participants self-reporting as African American and was performed at the Laboratory of Neurogenetics, National Institute on Aging, National Institutes of Health. Genotype data (for up to 907,763 SNPs) were generated for 1,024 participants using either Illumina 1M and 1M duo arrays (n=709) ,or a combination of 550K, 370K, 510S and 240S to equate the million SNP level of coverage. Inclusion criteria for genetic data in HANDLS includes concordance between self-reported sex and sex estimated from X chromosome heterogeneity, > 95% call rate per participant (across all equivalent arrays), concordance between self-reported African ancestry and ancestry confirmed by analyses of genotyped SNPs, and no cryptic relatedness to any other samples at a level of proportional sharing of genotypes > 15% (effectively excluding 1st cousins and closer relatives from the set of probands used in analyses). In addition, SNPs included in the analysis were filtered for HWE p-value > 1e-7, missing by haplotype p-values > 1e-7, minor allele frequency > 0.01, and call rate > 95%. Data analyses utilized the high-performance computational capabilities of the Biowulf Linux cluster at the NIH, Bethesda, Md. (http://biowulf.nih.gov).

INTERSTROKE-African Americans

INTERSTROKE is an international, multi-centered, case-control study of stroke investigating the global burden of risk factors across 32 countries and 18 different ethnic groups around the world. A detailed report of the study design has been published (Neuroepidemiology. 2010; 35:36-44). Briefly, cases were patients with acute first stroke (within 5 days of symptoms onset and 72 hours of hospital admission) in whom neuroimaging (CT or MRI) was performed. The TOAST classification system was used to define ischemic stroke subtypes. Cases were excluded if 1) they were unable to communicate due to severe stroke without a valid surrogate respondent (e.g. first-degree relative or spouse), 2) they were hospitalized for acute coronary syndrome/myocardial infarction, or 3) stroke was attributed to non-vascular causes (e.g. tumor). Controls were selected from the community and had no history of stroke.

A subset of INTERSTROKE participants consenting to genetic analysis with sufficient DNA quantities were genotyped on the Illumina Infinium Cardiometabo BeadChip. All samples were genotyped at a central site (the Genetic Molecular Epidemiology Laboratory in Hamilton, Ontario, Canada). Samples were excluded if they had 1) a high proportion of missing variants (missingness > 0.05), 2) inconsistencies between reported and genetically determined sex or ethnicity or 3) exhibited cryptic relatedness. Genotyped variants were excluded if they were rare (MAF < 0.01), exhibited high missingness across samples (missingness > 0.01), or deviated from hardy-weinberg equilibrium (P<5x10-6). Pre-phasing and imputation were perforrmed with SHAPEIT2 and IMPUTE, respectively, using the 1000Genomes Phase 1 Version 3 (November 23, 2010 subversion) reference panel. Imputed variants were removed if they were rare (MAF < 0.01) or of poor quality (INFO SCORE < 0.30).

Ischemic Stroke Genetic Study (ISGS)-African Americans

ISGS is a multicenter inception cohort study. Cases were recruited from inpatient stroke services at five United States academic medical centers. Cases are adult men and women over the age of 18 years diagnosed with first-ever ischemic stroke confirmed by a study neurologist on the basis of history, physical examination and CT or MR imaging of the brain. Cases had to be enrolled within 30 days of onset of stroke symptoms. Cases were excluded if they had: a mechanical aortic or mitral valve at the time of the index ischemic stroke, central nervous system vasculitis, or bacterial endocarditis. They were also excluded if they were known to have: cerebral autosomal dominant arteriopathy with subcortical infarcts and leukoencephalopathy (CADASIL), Fabry disease, homocystinuria, mitochondrial encephalopathy with lactic acidosis and stroke-like episodes (MELAS), or sickle cell anemia. Diagnostic evaluation included: head CT (95%) or MRI (83%), electrocardiography (92%), cervical arterial imaging (86%), and echocardiography (74%). Medical records from all cases were centrally reviewed by a vascular neurology committee and assigned ischemic stroke subtype diagnoses according to TOAST criteria, Oxfordshire Community Stroke the Baltimore-Washington Young Stroke Stud. DNA was donated to the NINDS DNA Repository (Coriell Institute, Camden, NJ) for eligible samples with appropriate written informed consent. DNA samples were genotyped using the Illumina 610 array and data analyses were supported by the high-performance computational capabilities of the Biowulf Linux cluster at the NIH (http://biowulf.nih.gov).

Jackson Heart Study (JHS)

The JHS is a single-site, prospective, population-based study designed to explore the environmental, behavioral, and genetic factors that influence the development of CVD among African Americans. A total of 5,301 women and men between the ages of 21 and 94 were recruited between 2000 and 2004 from a tri-county area of Mississippi: Hinds, Madison, and Rankin Counties. Participants were recruited from four sources, including (1) randomly sampled households from a commercial listing; (2) ARIC participants; (3) a structured volunteer sample that was designed to mirror the eligible population; and (4) a nested family cohort. Overviews of the JHS including the sampling and recruitment, sociocultural, and laboratory methods have been described and published previously.41-44 The institutional review boards of the following participating institutions approved the study: the University of Mississippi Medical Center, Jackson State University, and Tougaloo College. All of the participants provided written informed consent. Unrelated participants were between 35 and 84 years old, and members of the family cohort were ≥ 21 years old when consent for genetic testing was obtained and blood was drawn for DNA extraction.

The baseline examination consisted of a home interview, self-administered questionnaires, and a clinic visit. Medications taken in the prior 2 weeks were brought to clinic and transcribed verbatim with subsequent coding by a pharmacist. After an overnight fast, anthropometric and seated blood pressure measurements were obtained and venipuncture/urine collection was performed in accordance with the National Committee for Clinical Laboratory Standards. Blood pressure was measured by trained technicians using a Hawksley random zero manometer and determined by the arithmetic average of two readings taken 1 minute apart after a five-minute rest.45

Stroke Assessment in the JHS: In addition to the standard JHS examinations, participants were contacted by telephone annually beginning in 2005 to obtain interim information about cardiovascular events. (ICD-9 code 428 for hospitalizations). During the annual follow up phone call, participants or designated representative provide self-reported information of hospitalization or death. Identification and abstraction of CVD illness and death data are performed by a certified medical record abstractor. Incident stroke is defined as stroke that occurred while the participants was enrolled the study, i.e. stroke event occurred after the baseline visit. Strokes are classified as either definite or probable stroke. The definition of stroke was based on the World Health Organization (WHO) criteria for definition of stroke or clinical criteria in which case the WHO criteria might not have been satisfied, but there is clinical evidence sufficient for a diagnosis of stroke to be made. More details on identification and classification of stroke events in the JHS have already been published.56,57 Although not directly relevant in this study, ischemic stroke subtyping in the JHS was done the TOAST classification criteria.

The Sea Islands Genetics Network (SIGNET) & REasons for Geographic And Racial Differences in Stroke (REGARDS) – African Americans

The Sea Islands Genetics Network (SIGNET) study consists of the REasons for Geographic And Racial Differences in Stroke (REGARDS), the Sea Islands Genetic African American Registry (Project SuGAR), a COBRE for Oral Health study (COBRE), and the Systemic Lupus Erythematosus in Gullah Health study (SLEIGH). All subjects are African Americans (AA), and all provided written informed consent.

All SIGNET samples (n= 4,298) were genotyped using the Affymetrix Genome-Wide Human SNP Array 6.0. Imputation was performed using MACH (version 1.0.16) to impute all autosomal SNPs using the CEU+YRI reference panel (as supplied by Goncalo Abecasis) from build 36 (2,318,207 SNPs in total).

REGARDS is an observational cohort of 30,239 AA and white men and women enrolled in their homes after a telephone interview in 2003-7 (Howard VJ et al., 2005). Participants were a national sample oversampled from the southeastern stroke belt (56%) and were 58% female and 42% black by design. Participants were followed every 6 months by telephone to ascertain health outcomes, with validation of stroke, coronary heart disease, death and other ancillary study endpoints. For SIGNET, we selected all AA REGARDS type 2 diabetes (T2D) cases recruited from SC, GA, NC, and AL, and an equivalent number of race, sex, and age-strata matched diabetes-free controls. We also included all participants not already included that were current residents of the 15-county “Low Country” region of SC and GA (SC counties Beaufort, Berkeley, Charleston, Colleton, Dorchester, Georgetown, Hampton, Horry, Jasper; GA counties Bryan, Camden, Chatham, Glynn, Liberty, McIntosh). The subset of REGARDS participants genotyped under SIGNET are referred to as SIGNET-REGARDS. GWAS genotyping was completed among 2398 SIGNET-REGARDS AA participants, including 1149 with diabetes and 1249 without diabetes.

Siblings with Ischemic Stroke Study (SWISS) – African Americans

SWISS is a prospective multicenter affected sibling pair study of first-ever or recurrent ischemic stroke. Subjects were recruited from 54 enrolling hospitals across the US and Canada. Samples were collected between 1999-2011. Ischemic stroke probands were enrolled at 66 US medical centers and 4 Canadian medical centers. All recruits were extensively clinically phenotyped and have imaging- confirmed ischemic stroke using either CT or MRI brain scans. Probands are adult men and women over the age of 18 years diagnosed with ischemic stroke confirmed by a study neurologist on the basis of history, physical examination and CT or MR imaging of the brain who also have a history of at least one living sibling with a history of stroke. Probands were excluded if 1) they had a mechanical aortic or mitral valve at the time of the index ischemic stroke, central nervous system vasculitis, or bacterial endocarditis or 2) were known to have cerebral autosomal dominant arteriopathy with subcortical infarcts and leukoencephalopathy (CADASIL), Fabry disease, homocystinuria, mitochondrial encephalopathy with lactic acidosis and stroke-like episodes (MELAS), or sickle cell anemia. Siblings were enrolled using proband- initiated direct contact when permitted by Institutional Review Boards. Concordant siblings had their diagnosis of ischemic stroke confirmed by review of medical records by a central vascular neurology committee. Concordant siblings had the same eligibility criteria as probands. Subtype diagnoses were assigned to the index strokes of probands and concordant siblings according to TOAST criteria. Discordant siblings of the proband were confirmed to be stroke-free using the Questionnaire for Verifying Stroke-free Status. DNA samples were genotyped using the Illumina 660 array and data analyses were supported by the high-performance computational capabilities of the Biowulf Linux cluster at the NIH (http://biowulf.nih.gov).

Women’s Health Initiative (WHI) – African Americans

The goal of the WHI was to investigate the etiology and prevention of chronic disease in post-menopausal women . Approximately 161,000 postmenopausal women 50–79 years of age from 40 clinical centers in the US were recruited between 1993 and 1998. WHI consists of an observational study (OS), and clinical trials (CT) of postmenopausal hormone therapy (estrogen alone or estrogen plus progestin), a calcium and vitamin D supplement trial, and a dietary modification trial. A subset of 8,515 African American women who provided consent for DNA analysis were randomly selected for genome-wide genotyping as part of the SNP Health Association Resource (SHARe).

Genetic data were obtained from genome-wide scans using the Genome-wide Human SNP Array 6.0 (Affymetrix, Santa Clara, CA, www.affymetrix.com) of 909,622 single nucleotide polymorphisms (SNPs). Genotyping quality control included examination of concordance rates for blinded and un-blinded duplicates. Approximately 1% of SNPs failed genotyping and SNPs with call rates < 95% or concordance rates <98%, or minor allele frequency <1% were excluded. In addition to the genotyping, SNPs were imputed using 1000 Genomes Project phase 1 integrated variant set (Aug 2012). Principal components were calculated for each individual and evaluated for their contribution to ancestral variation. Because most of the ancestral variation was explained by the first 4 PCs, only these were included as covariates in the analyses.

Stroke ascertainment in WHI: All incident strokes, other vascular events, and deaths were identified through self-report at annual (OS) and semi-annual (CT) participant contacts, and through third- party reports by family members and proxies. Medical records were obtained for potential strokes, and adjudication was performed by trained physician adjudicators who assigned a diagnosis. Stroke diagnosis requiring and/or occurring during hospitalization was based on rapid onset of a neurological deficit attributable to an obstruction or rupture of an arterial vessel system. The deficit was not known to be secondary to brain trauma, tumor, infection or other cause and must have lasted more than 24 hours unless death supervened or a lesion compatible with acute stroke was evident on computed tomography or magnetic resonance imaging were classified as ischemic, hemorrhagic or unknown/missing. Ischemic stroke subtypes were further classified using Trial of Org 10172 in Acute Stroke Treatment (TOAST) analyses, strokes subtypes judged as ‘probable’ or ‘possible’ were combined. African American women passing the above genotyping quality control criteria, with follow-up data, and without a history of stroke at baseline were included in the WHI analyses. All participants provided written, informed consent.

SiGN

The Stroke Genetics Network (SiGN) study was funded by a cooperative agreement grant from the National Institute of Neurological Disorders and Stroke (NINDS) U01 NS069208. Genotyping services were provided by the Johns Hopkins University Center for Inherited Disease Research (CIDR), which is fully funded through a federal contract from the National Institutes of Health (NIH) to the Johns Hopkins University (contract No.HHSN268200782096C). The Biostatistics Department Genetics Coordinating Center at the University of Washington (Seattle) provided more extensive quality control of the genotype data through a subcontract with CIDR. Additional support to the Administrative Core of SiGN was provided by the Dean’s Office, University of Maryland School of Medicine. SiGN- Group 4 consists of AA subjects from the GASROS, GCNKSS, ISGS, MCISS, MIAMISR, NOMAS, REGARDS, SPS3, SWISS, WHI, and WUSTL studies. MGH-GASROS: The Massachusetts General Hospital Stroke Genetics Group was supported by the NIH Genes Affecting Stroke Risks and Outcomes Study (GASROS) grant K23 NS042720, the American Heart Association/Bugher Foundation Centers for Stroke Prevention Research 0775010N, and NINDS K23NS042695, K23 NS064052, the Deane Institute for Integrative Research in Atrial Fibrillation and Stroke, and by the Keane Stroke Genetics Fund. Genotyping services were provided by the Broad Institute Center for Genotyping and Analysis, supported by grant U54 RR020278 from the National Center for Research Resources. GCNKSS: The Greater Cincinnati/Northern Kentucky Stroke Study (GCNKSS) was supported by the NIH (NS030678). MCISS: The Middlesex County Ischemic Stroke Study (MCISS) was supported by intramural funding from the New Jersey Neuroscience Institute/JFK Medical Center, Edison, NJ, and The Neurogenetics Foundation, Cranbury, NJ. We acknowledge Dr Souvik Sen for his advice and encouragement in the initiation and design of this study. MIAMISR and NOMAS: The Northern Manhattan Study (NOMAS) was supported by grants from the NINDS (R37 NS029993, R01 NS27517). The Cerebrovascular Biorepository at University of Miami/Jackson Memorial Hospital (The Miami Stroke Registry, Institutional Review Board No. 20070386) was supported by the Department of Neurology at University of Miami Miller School of Medicine and Evelyn McKnight Brain Institute. Biorepository and DNA extraction services were provided by the Hussmann Institute for Human Genomics at the Miller School of Medicine. SPS3: The Secondary Prevention of Small Subcortical Strokes trial was funded by the US National Institute of Health and Neurological Disorders and Stroke grant No. U01NS38529- 04A1 (principal investigator, Oscar R. Benavente; coprincipal investigator, Robert G. Hart). The SPS3 Genetic Substudy (SPS3-GENES) was funded by R01 NS073346 (coprincipal investigators, Julie A. Johnson, Oscar R. Benavente, and Alan R. Shuldiner) and U01 GM074492-05S109 (principal investigator, Julie A. Johnson). WUSTL: Washington University St. Louis Stroke Study (WUSTL): The collection, extraction of DNA from blood, and storage of specimens were supported by 2 NINDS NIH grants (P50 NS055977 and R01 NS8541901). Basic demographic and clinical characterization of stroke phenotype was prospectively collected in the Cognitive Rehabilitation and Recovery Group (CRRG) registry. The Recovery Genomics after Ischemic Stroke (ReGenesIS) study was supported by a grant from the Barnes-Jewish Hospital Foundation.

The descriptions in this section are adopted from the GIGASTROKE <https://www.nature.com/articles/s41586-022-05165-3>

REFERENCES

1 Traylor, M. *et al.* Genetic risk factors for ischaemic stroke and its subtypes (the METASTROKE collaboration): a meta-analysis of genome-wide association studies. *Lancet Neurol* **11**, 951-962, doi:10.1016/s1474-4422(12)70234-x (2012).

2 Neurology Working Group of the Cohorts for Heart and Aging Research in Genomic Epidemiology (CHARGE) Consortium, t. S. G. N. S., and the International Stroke Genetics Consortium (ISGC);. Identification of additional risk loci for stroke and small vessel disease: a meta-analysis of genome-wide association studies. *Lancet Neurol* **15**, 695-707, doi:10.1016/s1474-4422(16)00102-2 (2016).

3 Psaty, B. M. *et al.* Cohorts for Heart and Aging Research in Genomic Epidemiology (CHARGE) Consortium: Design of prospective meta-analyses of genome-wide association studies from 5 cohorts. . *Circ Cardiovasc Genet* **2**, 273-280 (2009).

4 Harris, T. B. *et al.* Age, Gene/Environment Susceptibility-Reykjavik Study: multidisciplinary applied phenomics. *Am J Epidemiol* **165**, 1076-1087 (2007).

5 Fried, L. P. *et al.* The Cardiovascular Health Study: design and rationale. *Ann Epidemiol* **1**, 263-276, doi:10.1016/1047-2797(91)90005-w (1991).

6 Longstreth, W. T., Jr. *et al.* Frequency and predictors of stroke death in 5,888 participants in the Cardiovascular Health Study. *Neurology* **56**, 368-375 (2001).

7 Price, T. R., Psaty, B., O'Leary, D., Burke, G. & Gardin, J. Assessment of cerebrovascular disease in the Cardiovascular Health Study. *Ann Epidemiol* **3**, 504-507 (1993).

8 SHEP_Cooperative_Research_Group. Prevention of stroke by antihypertensive drug treatment in older persons with isolated systolic hypertension. Final results of the Systolic Hypertension in the Elderly Program (SHEP). SHEP Cooperative Research Group. *JAMA* **265**, 3255-3264 (1991).

9 Adams, H. P. *et al.* Classification of subtype of acute ischemic stroke. Definitions for use in a multicenter clinical trial. TOAST. Trial of Org 10172 in Acute Stroke Treatment. *Stroke* **24**, 35-41, doi:10.1161/01.str.24.1.35 (1993).

10 Dawber, T. R. & Kannel, W. B. The Framingham study. An epidemiological approach to coronary heart disease. *Circulation* **34**, 553-555 (1966).

11 Feinleib, M., Kannel, W. B., Garrison, R. J., McNamara, P. M. & Castelli, W. P. The Framingham Offspring Study. Design and preliminary data. *Prev Med* **4**, 518-525 (1975).

12 Splansky, G. L. *et al.* The Third Generation Cohort of the National Heart, Lung, and Blood Institute's Framingham Heart Study: design, recruitment, and initial examination. *Am J Epidemiol* **165**, 1328-1335 (2007).

13 Carandang, R. *et al.* Trends in incidence, lifetime risk, severity, and 30-day mortality of stroke over the past 50 years. *JAMA* **296**, 2939-2946 (2006).

14 Seshadri, S. *et al.* The lifetime risk of stroke: estimates from the Framingham Study. *Stroke* **37**, 345-350 (2006).

15 Wolf, P. A., Kannel, W. B. & Dawber, T. R. Prospective investigations: the Framingham study and the epidemiology of stroke. *Adv Neurol* **19**, 107-120 (1978).

16 Vartiainen, E. *et al.* Thirty-five-year trends in cardiovascular risk factors in Finland. *Int J Epidemiol* **39**, 504-518, doi:10.1093/ije/dyp330 (2010).

17 Tolonen, H. *et al.* The validation of the Finnish Hospital Discharge Register and Causes of Death Register data on stroke diagnoses. *Eur J Cardiovasc Prev Rehabil* **14**, 380-385, doi:10.1097/01.hjr.0000239466.26132.f2 (2007).

18 Hofman, A. Recent trends in cardiovascular epidemiology. *Eur J Epidemiol* **24**, 721-723 (2009).

19 Pahor, M. & Kritchevsky, S. Research hypotheses on muscle wasting, aging, loss of function and disability. *J Nutr Health Aging* **2**, 97-100 (1998).

20 Cesari, M. *et al.* Inflammatory markers and onset of cardiovascular events: results from the Health ABC study. *Circulation* **108**, 2317-2322, doi:10.1161/01.cir.0000097109.90783.fc (2003).

21 Houston, D. K. *et al.* Dietary fat and cholesterol and risk of cardiovascular disease in older adults: the Health ABC Study. *Nutr Metab Cardiovasc Dis* **21**, 430-437, doi:10.1016/j.numecd.2009.11.007 (2011).

22 Hofman, A. *et al.* The Rotterdam Study: objectives and design update. *Eur J Epidemiol* **22**, 819-829 (2007).

23 Hofman, A. *et al.* The Rotterdam Study: 2010 objectives and design update. *Eur J Epidemiol* **24**, 553-572 (2009).

24 Hofman, A., Grobbee, D. E., de Jong, P. T. & van den Ouweland, F. A. Determinants of disease and disability in the elderly: the Rotterdam Elderly Study. *Eur J Epidemiol* **7**, 403-422 (1991).

25 Bots, M. L. *et al.* Prevalence of stroke in the general population. The Rotterdam Study. *Stroke* **27**, 1499-1501 (1996).

26 Hollander, M. *et al.* Incidence, risk, and case fatality of first ever stroke in the elderly population. The Rotterdam Study. *J Neurol Neurosurg Psychiatry* **74**, 317-321 (2003).

27 Volzke, H. *et al.* Cohort profile: the study of health in Pomerania. *Int J Epidemiol* **40**, 294-307.

28 Szentkiralyi, A., Volzke, H., Hoffmann, W., Happe, S. & Berger, K. A time sequence analysis of the relationship between cardiovascular risk factors, vascular diseases and restless legs syndrome in the general population. *J Sleep Res* **22**, 434-442, doi:10.1111/jsr.12040 (2013).

29 Ridker, P. M. *et al.* Rationale, design, and methodology of the Women's Genome Health Study: a genome-wide association study of more than 25,000 initially healthy american women. *Clin Chem* **54**, 249-255, doi:10.1373/clinchem.2007.099366 (2008).

30 Bild, D. E. *et al.* Multi-ethnic study of atherosclerosis: objectives and design. *Am J Epidemiol* **156**, 871-881 (2002).

31 Kawasaki, R. *et al.* Retinal microvascular signs and risk of stroke: the Multi-Ethnic Study of Atherosclerosis (MESA). *Stroke* **43**, 3245-3251, doi:10.1161/strokeaha.112.673335 (2012).

32 Shepherd, J. *et al.* Pravastatin in elderly individuals at risk of vascular disease (PROSPER): a randomised controlled trial. *Lancet* **360**, 1623-1630 (2002).

33 Shepherd, J. *et al.* The design of a prospective study of Pravastatin in the Elderly at Risk (PROSPER). PROSPER Study Group. PROspective Study of Pravastatin in the Elderly at Risk. *Am J Cardiol* **84**, 1192-1197 (1999).

34 3C-Study-Group. Vascular factors and risk of dementia: design of the Three-City Study and baseline characteristics of the study population. *Neuroepidemiology* **22**, 316-325 (2003).

35 Riboli, E. *et al.* European Prospective Investigation into Cancer and Nutrition (EPIC): study populations and data collection. *Public Health Nutr* **5**, 1113-1124, doi:10.1079/PHN2002394 (2002).

36 Danesh, J. *et al.* EPIC-Heart: the cardiovascular component of a prospective study of nutritional, lifestyle and biological factors in 520,000 middle-aged participants from 10 European countries. *Eur J Epidemiol* **22**, 129-141, doi:10.1007/s10654-006-9096-8 (2007).

37 InterAct, C. *et al.* Design and cohort description of the InterAct Project: an examination of the interaction of genetic and lifestyle factors on the incidence of type 2 diabetes in the EPIC Study. *Diabetologia* **54**, 2272-2282, doi:10.1007/s00125-011-2182-9 (2011).

38 Debette, S. *et al.* Common variation in PHACTR1 is associated with susceptibility to cervical artery dissection. *Nat Genet* **47**, 78-83, doi:10.1038/ng.3154 (2015).

39 Ay, H. *et al.* A computerized algorithm for etiologic classification of ischemic stroke: the Causative Classification of Stroke System. *Stroke* **38**, 2979-2984 (2007).

40 Carty, C. L. *et al.* Meta-Analysis of Genome-Wide Association Studies Identifies Genetic Risk Factors for Stroke in African Americans. *Stroke* **46**, 2063-2068, doi:10.1161/strokeaha.115.009044 (2015).
