# Supplementary material for: Novel functional insights into ischemic stroke biology provided by the first genome-wide association study of stroke in indigenous Africans
Source: Genome Med. 2024 Feb 5;16:25. doi: 10.1186/s13073-023-01273-5 (PMC10840175; doi:10.1186/s13073-023-01273-5)
Supplement: Supplementary file 3 — Additional file 3. Additional Discussion. [file 13073_2023_1273_MOESM3_ESM.docx]

**Additional Discussion**

[**Additional discussion point a** highlights the point that there are additional 30 SNPs with suggestive evidence of association in addition to the 2 with genomic significance]

Overall, 2 SNPs attained genomic significance in their association with ischemic stroke while additional 30 SNPs had suggestive evidence of association. In looking up variants previously associated with stroke in COMPASS, none of the 29 SNPs in COMPASS met the Bonferroni-threshold for significance in SIREN (P-values=0.05/29=0.0017).^1^

[**Additional discussion point b** highlights other marginally significant genetic variants]

Several marginally significant associations were observed with other SNPs associated with ischemic stroke in chromosome 2 which were more than 77kb from the closest gene *LINC01854* and SNPs in chromosome 7 which were more than 116kb to the closest gene *LINC01446* (P-value<1.00E-06). In addition, we observed marginally significant SNPs in genes *LINCO2052* (chromosome 3), *DGKG* (chromosome 3), *CLIC5* (chromosome 6) and *GALTN9* (Chromosome 12). (P-value<1.00E-06).

Genetic variants with suggestive association with ischemic stroke includes the region around *LINC01854* which is associated with the regulation of DNA methylation variation^2,3^ ^4^ ^5^ There were also SNPs in loci proximate to *POM121L12* gene which has been previously associated with smoking status^6^ and *LINC01446* is associated with pulse pressure^7^, and carotid atherosclerosis^8^ ^9^ ^10^.

Another intergenic SNP rs142793076 mapped to the region between *DGKG* and *LINC02052* was protectively associated with ischemic stroke in this study. The *DGKG* gene which is expressed in the brain encodes an enzyme involved in lipid metabolism and has been associated with body mass index^11^. *LINC02052* has been previously associated with functional outcomes in ischemic stroke^12^, and systolic blood pressure^13^ ^3^.

SNP rs147996143 is on *CLIC5,* which is associated with processes including (vascular) endothelial cell maintenance.^14,15^. SNP rs76534667 is near *GALNT9* which is expressed specifically in the brain and has been associated with multiple phenotypes including body mass index^16^ ^17^,^18^.

[**Additional discussion point c** highlights the relationships of directions of effect between associations of the loci with ischaemic stroke in SIREN, COMPASS and MEGASTROKE]

The similarity of direction of effect between associations of the loci with ischemic stroke in both SIREN and COMPASS studies strengthens the validity of the association of these loci with ischemic stroke (Supplementary Figure 4).^19^ Despite the similarity in direction of effects which strengthens some of the associations, none of the MEGASTROKE SNPs^20^ showed nominal association with the SIREN SNPs. This further reinforces the level of disparity in population linkage disequilibrium and allele frequency differences between the African–ancestry and European–ancestry populations in the SIREN and MEGASTROKE cohorts, respectively.

[**Additional discussion point d** provides further details on the non – transferability of African ancestry specific risk loci including other similar examples from other African studies]

The ideal replication cohort will be a large sample of indigenous African stroke patients with GWAS datasets ^21,22^ ^23,24^ but this is currently non-existent. Non-transferability of African ancestry specific risk loci in non-indigenous African populations has also been observed in African GWAS studies of other non-communicable diseases including Type 2 DM^19^, primary open angle glaucoma^25^ and dyslipidemia^26^. In a previous meta - analysis of primary open angle glaucoma GWAS among European, Asian and African ancestry populations, in the African meta-analysis, one locus (rs16944405 within *IQGAP1*) reached the genome-wide significance level (P-value = 3.0E-08). but demonstrated non - transferability to the European (P-value = 0.315) and Asian (P-value = 0.075) datasets suggesting that it may be an African - specific variant ^25^. Similarly, in a GWAS study of type 2 DM among sub-Saharan Africans, several SNPs in previous type 2 DM GWAS of predominantly European samples were not transferable to the African GWAS datasets ^19^. A recent meta-analysis of genetic associations for lipid traits also identified hundreds of variants with clear differences across European, Asian and African studies and several African ancestry - specific novel loci.^27^. The existence of such ancestry - specific variants have implications for the development of polygenic risk scores (PRS) of higher accuracies in the stratification of individuals based on disease risks. This therefore strengthens the argument for ancestry or region-specific PRS

.[**Additional discussion point e** highlights further details of the strengths of our study]

Our study has a major strength in being the first stroke GWAS in an indigenous African population. Furthermore, we performed an African ancestry meta-analysis involving an African American population and a trans–ancestry meta-analysis which further involved European ancestry populations. We also conducted fine mapping and gene expression studies in *silico* to functionally identify and better characterize identified key genetic variants. We further explored the genomic landscape surrounding the lead significant SNPs in our study through chromatin interaction and eQTL mapping. The study thus contributes to the characterization of ancestry- specific genetic risk factors of stroke with potential implications for risk assessment and identification of new molecular targets for prediction, prevention, diagnosis, prognosis, and treatment of stroke.

**References**

1 Keene, K. L. *et al.* Genome-Wide Association Study Meta-Analysis of Stroke in 22 000 Individuals of African Descent Identifies Novel Associations With Stroke. *Stroke* **51**, 2454-2463, doi:10.1161/STROKEAHA.120.029123 (2020).

2 Zhang, Q. *et al.* Genotype effects contribute to variation in longitudinal methylome patterns in older people. *Genome Med* **10**, 75, doi:10.1186/s13073-018-0585-7 (2018).

3 Wang, H. *et al.* Genome-wide interaction analysis of pathological hallmarks in Alzheimer's disease. *Neurobiol Aging* **93**, 61-68, doi:10.1016/j.neurobiolaging.2020.04.025 (2020).

4 Kapoor, M. *et al.* Genome-wide survival analysis of age at onset of alcohol dependence in extended high-risk COGA families. *Drug Alcohol Depend* **142**, 56-62, doi:10.1016/j.drugalcdep.2014.05.023 (2014).

5 Teumer, A. *et al.* Genome-wide association study of chronic periodontitis in a general German population. *J Clin Periodontol* **40**, 977-985, doi:10.1111/jcpe.12154 (2013).

6 Cai, N. *et al.* Minimal phenotyping yields genome-wide association signals of low specificity for major depression. *Nat Genet* **52**, 437-447, doi:10.1038/s41588-020-0594-5 (2020).

7 Wain, L. V. *et al.* Novel Blood Pressure Locus and Gene Discovery Using Genome-Wide Association Study and Expression Data Sets From Blood and the Kidney. *Hypertension*, doi:10.1161/HYPERTENSIONAHA.117.09438 (2017).

8 Franceschini, N. *et al.* GWAS and colocalization analyses implicate carotid intima-media thickness and carotid plaque loci in cardiovascular outcomes. *Nat Commun* **9**, 5141, doi:10.1038/s41467-018-07340-5 (2018).

9 Kurilshikov, A. *et al.* Large-scale association analyses identify host factors influencing human gut microbiome composition. *Nat Genet* **53**, 156-165, doi:10.1038/s41588-020-00763-1 (2021).

10 Ravenhall, M. *et al.* Novel genetic polymorphisms associated with severe malaria and under selective pressure in North-eastern Tanzania. *PLoS Genet* **14**, e1007172, doi:10.1371/journal.pgen.1007172 (2018).

11 Wojcik, G. L. *et al.* Genetic analyses of diverse populations improves discovery for complex traits. *Nature* **570**, 514-518, doi:10.1038/s41586-019-1310-4 (2019).

12 Soderholm, M. *et al.* Genome-wide association meta-analysis of functional outcome after ischemic stroke. *Neurology* **92**, e1271-e1283, doi:10.1212/WNL.0000000000007138 (2019).

13 Kichaev, G. *et al.* Leveraging Polygenic Functional Enrichment to Improve GWAS Power. *Am J Hum Genet* **104**, 65-75, doi:10.1016/j.ajhg.2018.11.008 (2019).

14 Khawaja, A. P. *et al.* Genome-wide analyses identify 68 new loci associated with intraocular pressure and improve risk prediction for primary open-angle glaucoma. *Nat Genet* **50**, 778-782, doi:10.1038/s41588-018-0126-8 (2018).

15 Holliday, E. G. *et al.* Insights into the genetic architecture of early stage age-related macular degeneration: a genome-wide association study meta-analysis. *PLoS One* **8**, e53830, doi:10.1371/journal.pone.0053830 (2013).

16 Locke, A. E. *et al.* Genetic studies of body mass index yield new insights for obesity biology. *Nature* **518**, 197-206, doi:10.1038/nature14177 (2015).

17 Lencer, R. *et al.* Genome-wide association studies of smooth pursuit and antisaccade eye movements in psychotic disorders: findings from the B-SNIP study. *Transl Psychiatry* **7**, e1249, doi:10.1038/tp.2017.210 (2017).

18 Galvan-Femenia, I. *et al.* Multitrait genome association analysis identifies new susceptibility genes for human anthropometric variation in the GCAT cohort. *J Med Genet* **55**, 765-778, doi:10.1136/jmedgenet-2018-105437 (2018).

19 Adeyemo, A. A. *et al.* ZRANB3 is an African-specific type 2 diabetes locus associated with beta-cell mass and insulin response. *Nat Commun* **10**, 3195, doi:10.1038/s41467-019-10967-7 (2019).

20 Malik, R. *et al.* Multiancestry genome-wide association study of 520,000 subjects identifies 32 loci associated with stroke and stroke subtypes. *Nat Genet* **50**, 524-537, doi:10.1038/s41588-018-0058-3 (2018).

21 Studies, N.-N. W. G. o. R. i. A. *et al.* Replicating genotype-phenotype associations. *Nature* **447**, 655-660, doi:10.1038/447655a (2007).

22 Shriner, D. *et al.* Transferability and fine-mapping of genome-wide associated loci for adult height across human populations. *PLoS One* **4**, e8398, doi:10.1371/journal.pone.0008398 (2009).

23 Owolabi, M. *et al.* Advancing stroke genomic research in the age of Trans-Omics big data science: Emerging priorities and opportunities. *J Neurol Sci* **382**, 18-28, doi:10.1016/j.jns.2017.09.021 (2017).

24 Choudhury, A. *et al.* High-depth African genomes inform human migration and health. *Nature* **586**, 741-748, doi:10.1038/s41586-020-2859-7 (2020).

25 Gharahkhani, P. *et al.* Genome-wide meta-analysis identifies 127 open-angle glaucoma loci with consistent effect across ancestries. *Nat Commun* **12**, 1258, doi:10.1038/s41467-020-20851-4 (2021).

26 Choudhury, A. *et al.* Meta-analysis of sub-Saharan African studies provides insights into genetic architecture of lipid traits. *Nat Commun* **13**, 2578, doi:10.1038/s41467-022-30098-w (2022).

27 Choudhury, A. *et al.* Author Correction: Meta-analysis of sub-Saharan African studies provides insights into genetic architecture of lipid traits. *Nat Commun* **13**, 4474, doi:10.1038/s41467-022-32072-y (2022).
