# Supplementary material for: Novel functional insights into ischemic stroke biology provided by the first genome-wide association study of stroke in indigenous Africans
Source: Genome Med. 2024 Feb 5;16:25. doi: 10.1186/s13073-023-01273-5 (PMC10840175; doi:10.1186/s13073-023-01273-5)
Supplement: Supplementary file 4 — Additional file 4: Fig. S1. PC1 vs. PC2 plot of genotypes from SIREN and 1000G populations. Fig. S2. Manhattan Plots. Fig. S3. Locus zoom plots for SNPs rs112549349 (Chr. 2), rs147996143 (Chr. 6), rs2194650 (Chr. 7), rs76534667 (Chr. 12), and rs7326843 (Chr. 13). Fig. S4. Scatter plot showing the direction of effect (beta values) between associations of the SNPs with ischemic stroke in both SIREN and COMPASS studies. Fig. S5. Comparison of Minor Allele Frequencies among SIREN, COMPASS, and MEGASTROKE. Fig. S6a. Tissue gene expression for MIR4458HG using GTExPortal V8. Fig. S6b. Tissue gene expression for AADACL2 using GTExPortal V8. Fig. S7. (a) FUMA GENE2FUNC differentially expressed genes (DEG) output for 30 general tissue types. (b) FUMA GENE2FUNC differentially expressed genes (DEG) output for 54 specific tissue types. Red bars denote significantly enriched DEG sets (Bonferroni adjusted P-values). [file 13073_2023_1273_MOESM4_ESM.pptx]

## Slide 1
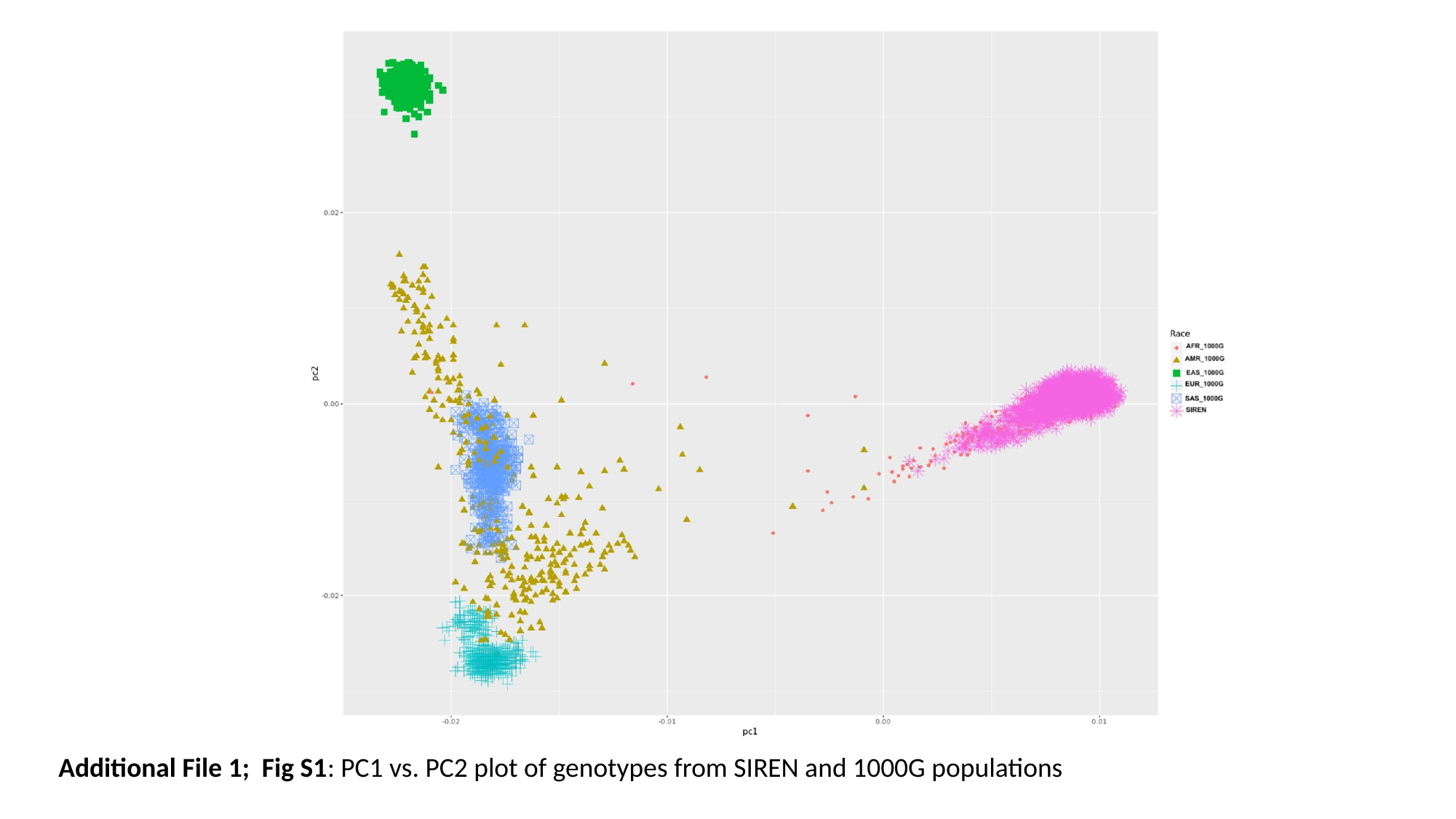

Additional File 1; Fig S1: PC1 vs. PC2 plot of genotypes from SIREN and 1000G populations

## Slide 2
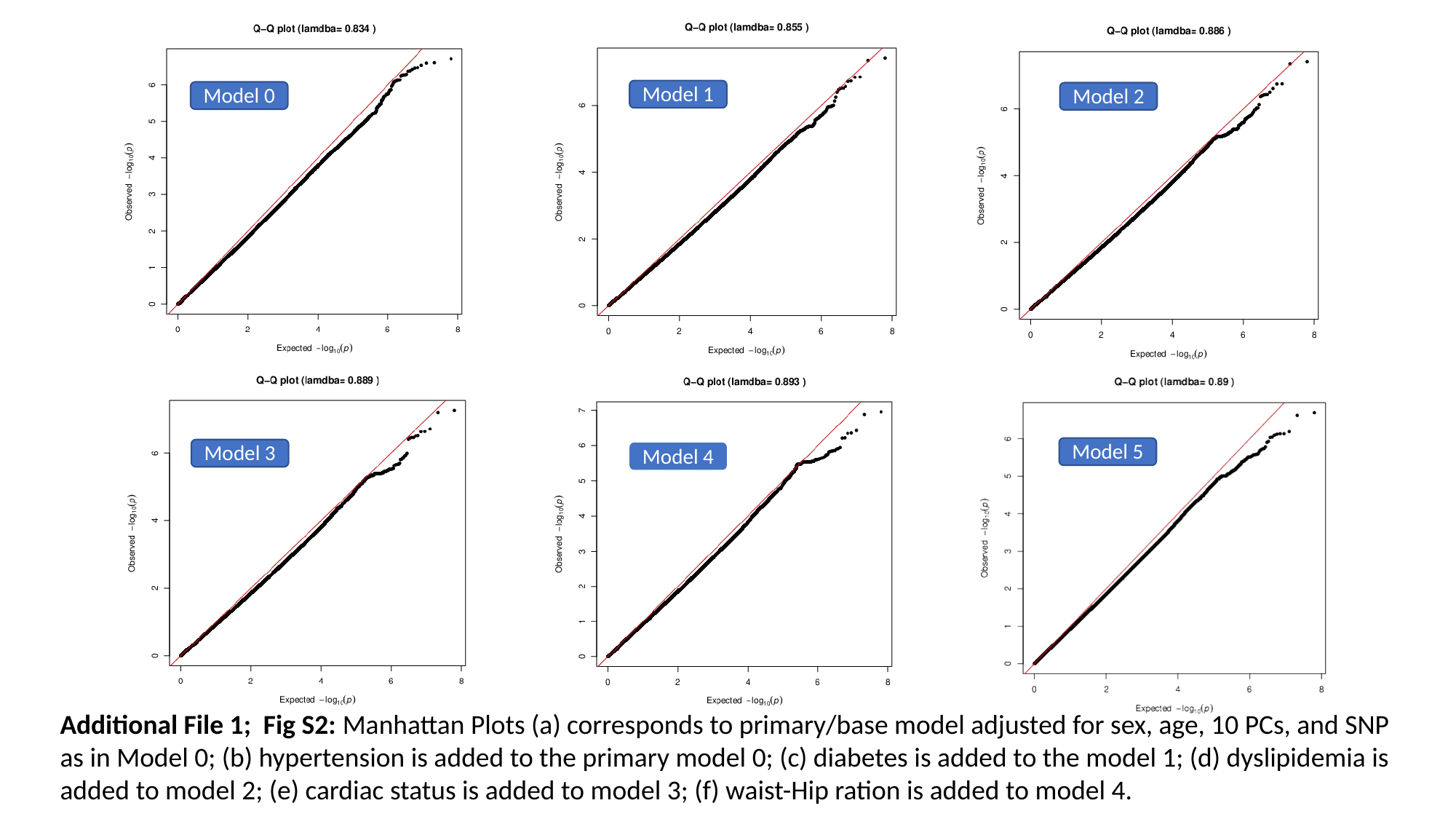

Model 1
Model 0
Model 2
Model 5
Model 3
Model 4
Additional File 1; Fig S2: Manhattan Plots (a) corresponds to primary/base model adjusted for sex, age, 10 PCs, and SNP as in Model 0; (b) hypertension is added to the primary model 0; (c) diabetes is added to the model 1; (d) dyslipidemia is added to model 2; (e) cardiac status is added to model 3; (f) waist-Hip ration is added to model 4.

## Slide 3
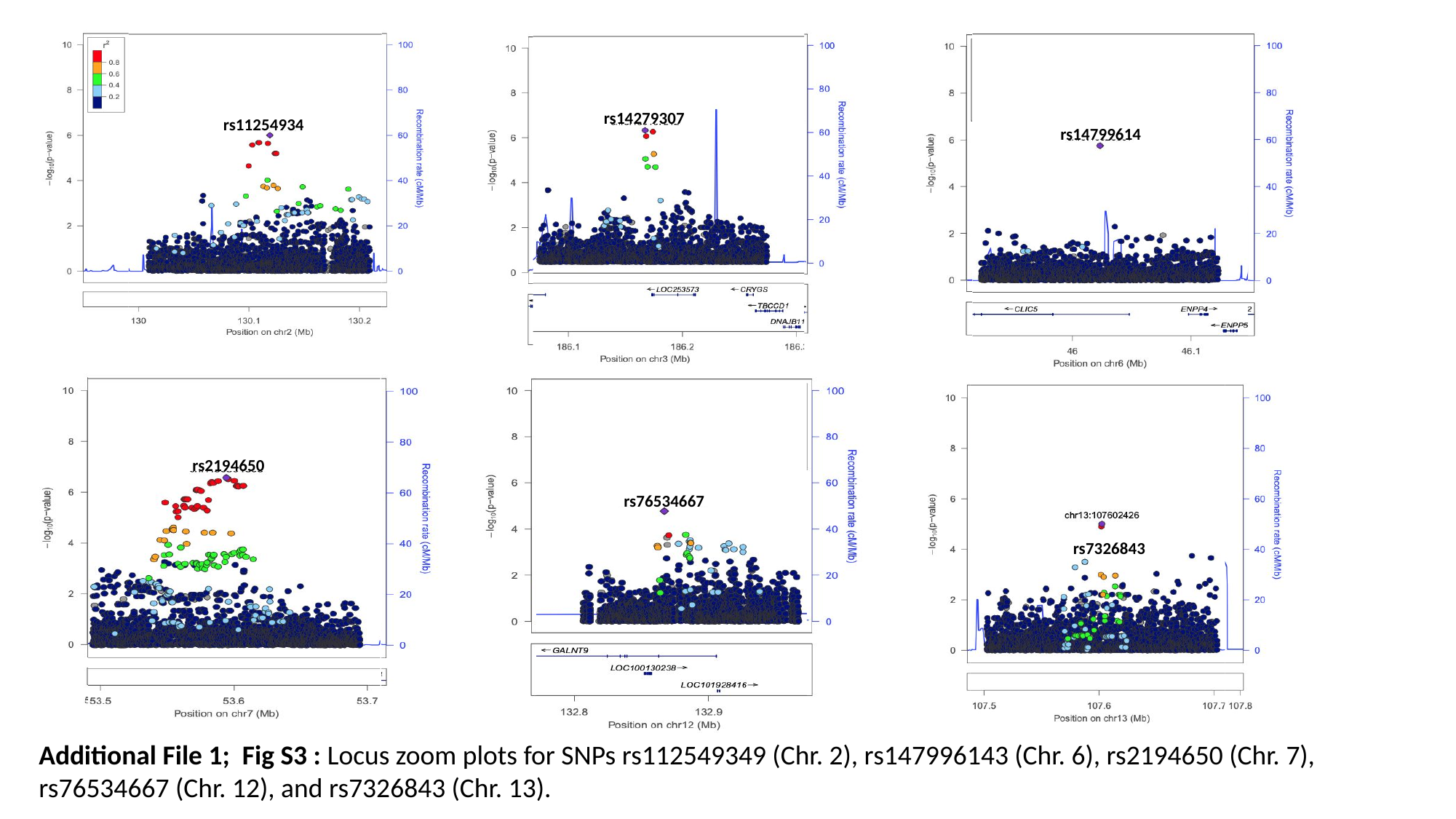

rs14279307
rs14799614
rs11254934
rs76534667
rs2194650
rs7326843
Additional File 1; Fig S3 : Locus zoom plots for SNPs rs112549349 (Chr. 2), rs147996143 (Chr. 6), rs2194650 (Chr. 7), rs76534667 (Chr. 12), and rs7326843 (Chr. 13).

## Slide 4
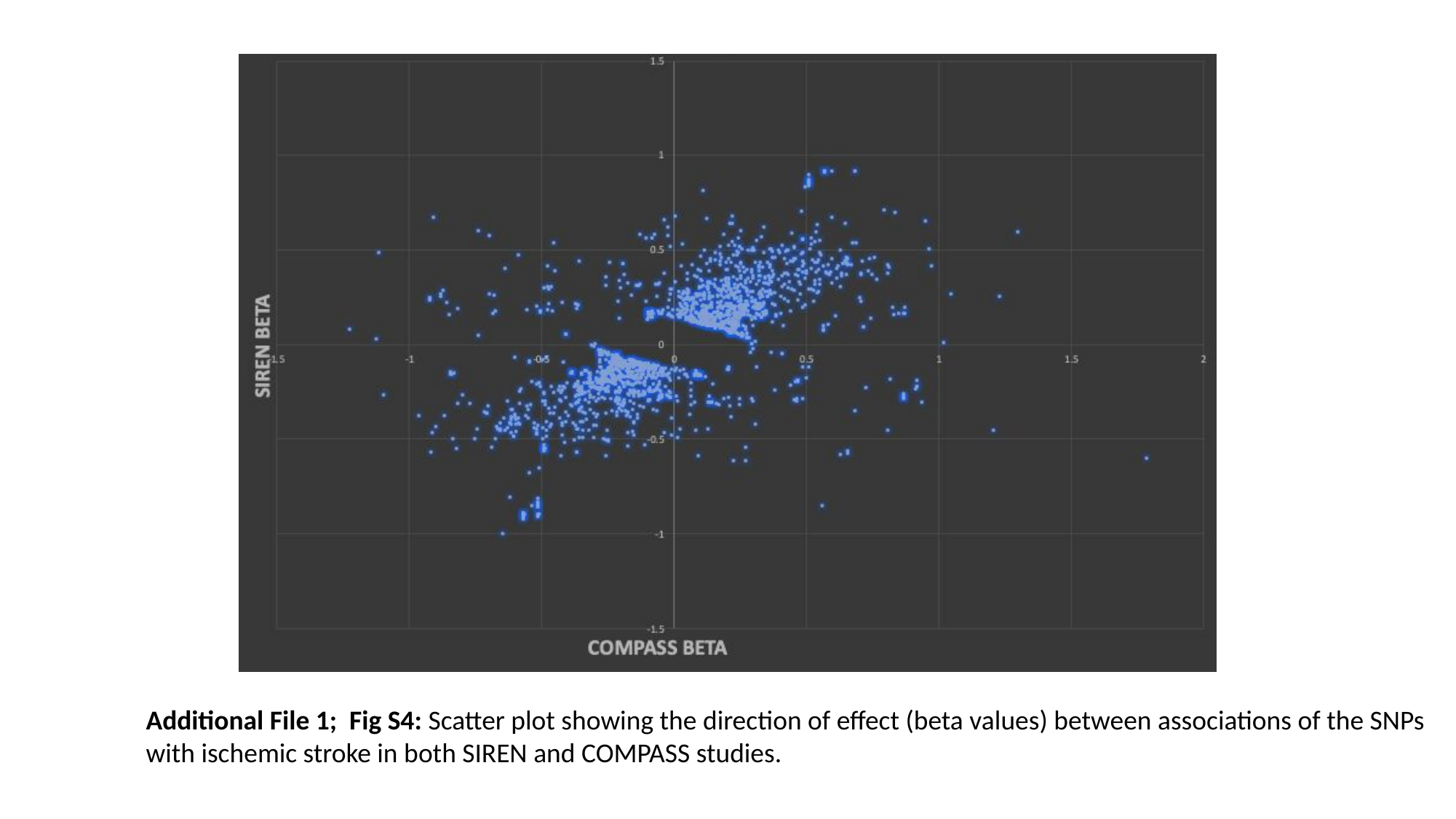

Additional File 1; Fig S4: Scatter plot showing the direction of effect (beta values) between associations of the SNPs with ischemic stroke in both SIREN and COMPASS studies.

## Slide 5
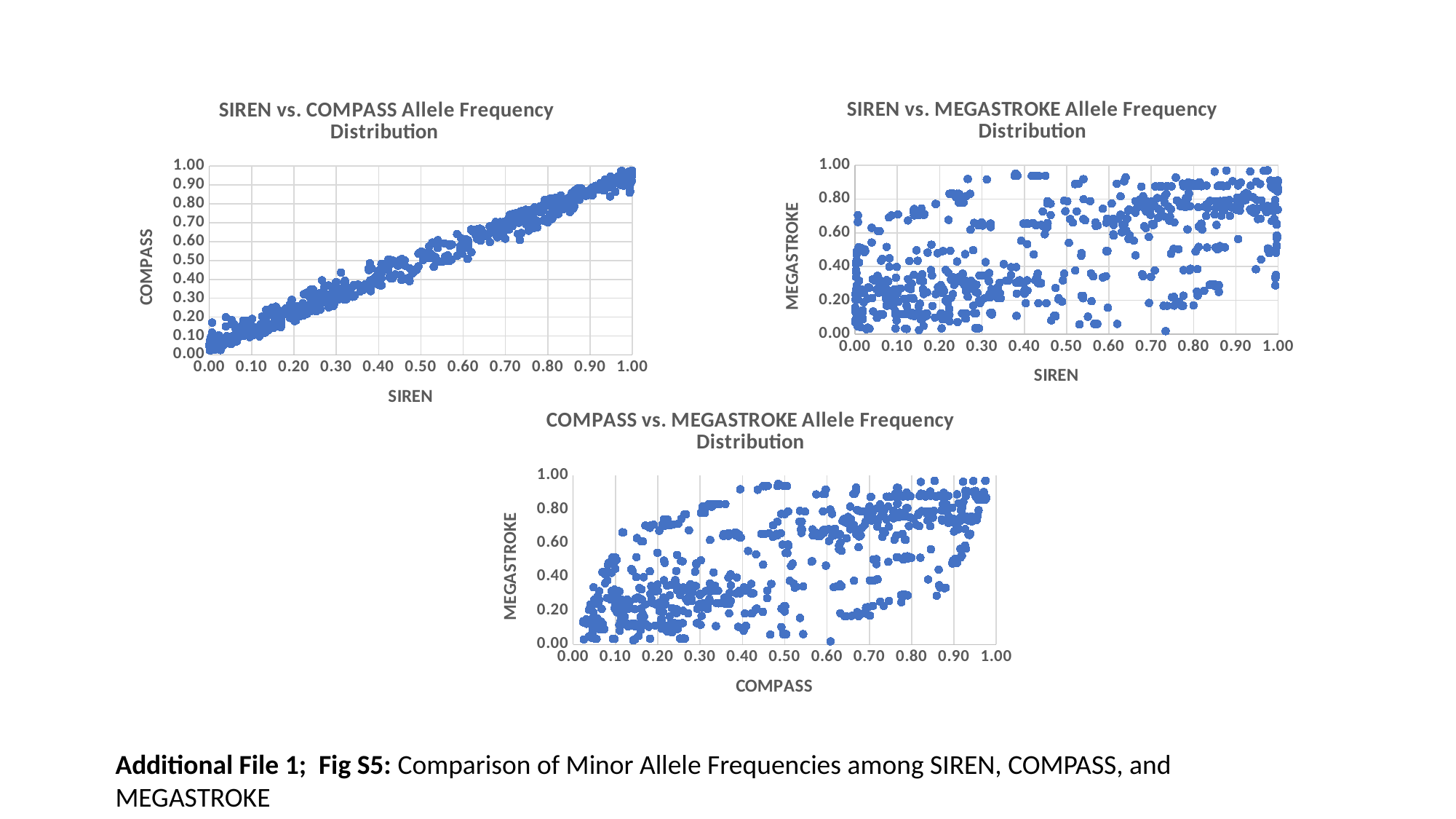

### Chart: SIREN vs. COMPASS Allele Frequency Distribution
| Category | COMPASS |
|---|---|
### Chart: SIREN vs. MEGASTROKE Allele Frequency Distribution
| Category | MEGASTROKE |
|---|---|
### Chart: COMPASS vs. MEGASTROKE Allele Frequency Distribution
| Category | MEGASTROKE |
|---|---|Additional File 1; Fig S5: Comparison of Minor Allele Frequencies among SIREN, COMPASS, and MEGASTROKE

## Slide 6
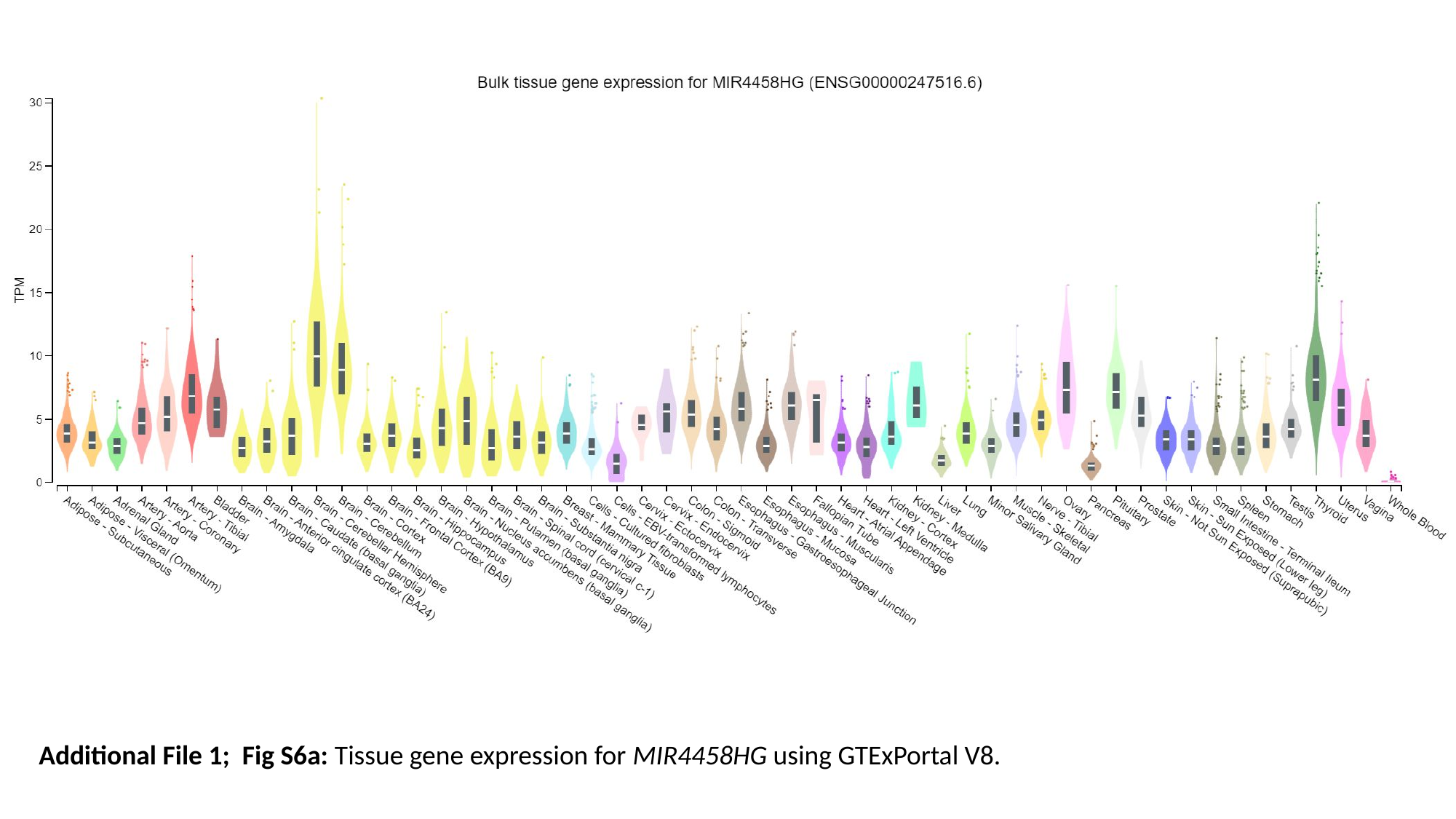

Additional File 1; Fig S6a: Tissue gene expression for MIR4458HG using GTExPortal V8.

## Slide 7
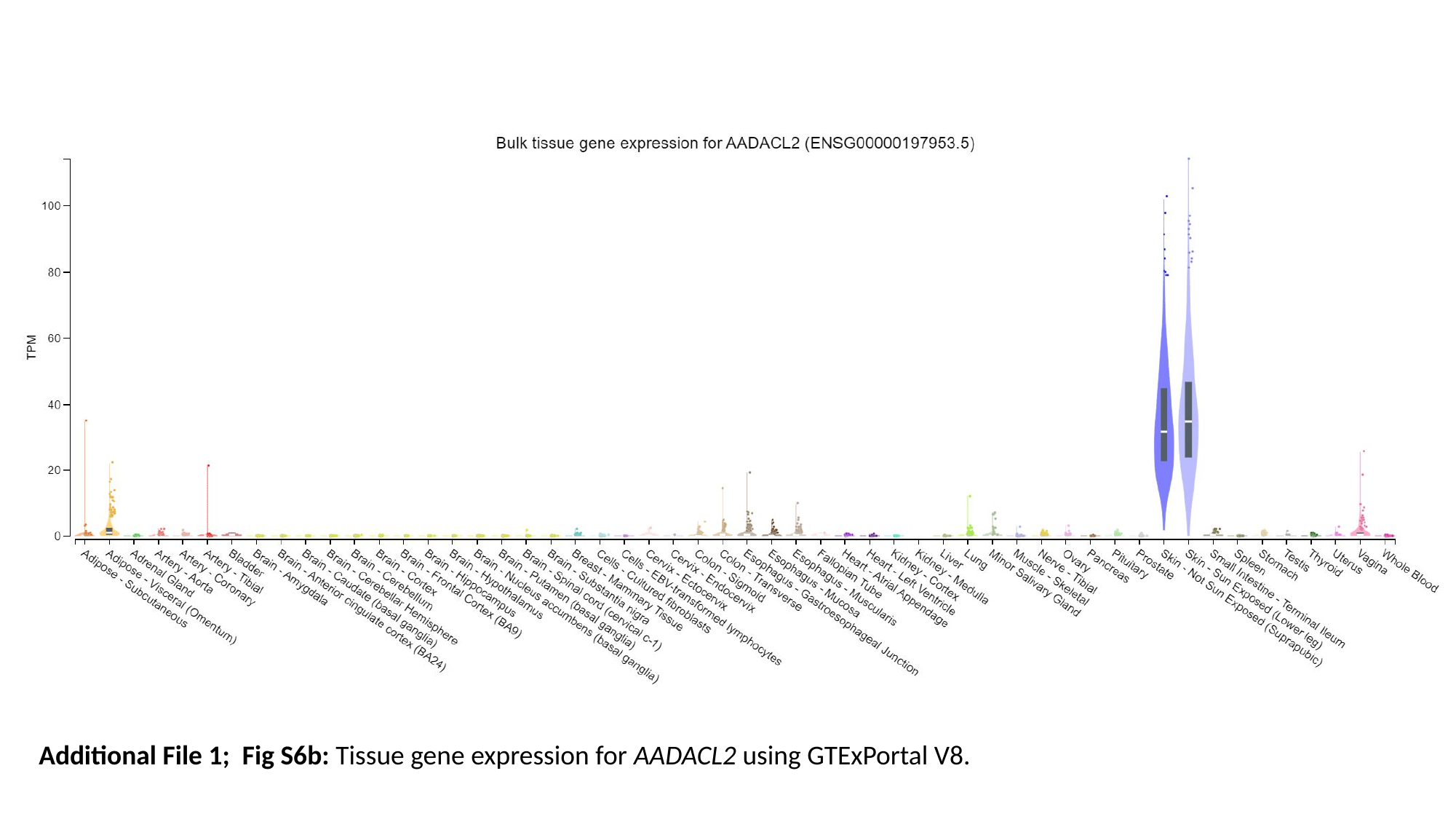

Additional File 1; Fig S6b: Tissue gene expression for AADACL2 using GTExPortal V8.

## Slide 8
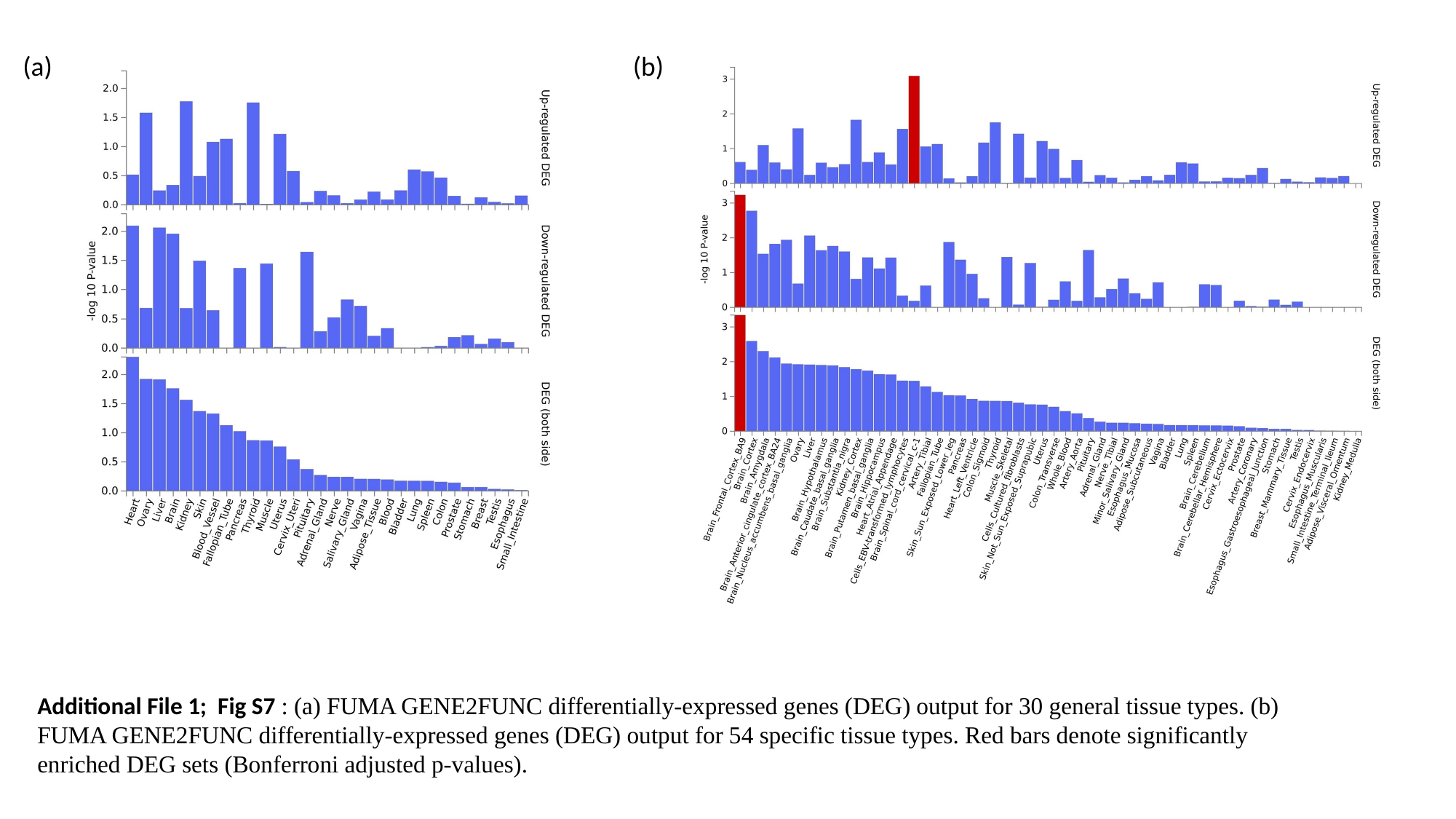

(a)
(b)
Additional File 1; Fig S7 : (a) FUMA GENE2FUNC differentially-expressed genes (DEG) output for 30 general tissue types. (b) FUMA GENE2FUNC differentially-expressed genes (DEG) output for 54 specific tissue types. Red bars denote significantly enriched DEG sets (Bonferroni adjusted p-values).
